# Variation in the Oral Processing of Everyday Meals Is Associated with Fullness and Meal Size; A Potential Nudge to Reduce Energy Intake?

**DOI:** 10.3390/nu8050315

**Published:** 2016-05-21

**Authors:** Danielle Ferriday, Matthew L. Bosworth, Nicolas Godinot, Nathalie Martin, Ciarán G. Forde, Emmy Van Den Heuvel, Sarah L. Appleton, Felix J. Mercer Moss, Peter J. Rogers, Jeffrey M. Brunstrom

**Affiliations:** 1 Nutrition and Behaviour Unit, School of Experimental Psychology, University of Bristol, Bristol BS8 1TU, UK; m_bosworth@hotmail.co.uk (M.L.B.); emmyheuvel@gmail.com (E.V.D.H.); sarahapple01@hotmail.com (S.L.A.); F.MercerMoss@bristol.ac.uk (F.J.M.M.); Peter.Rogers@bristol.ac.uk (P.J.R.); Jeff.Brunstrom@bristol.ac.uk (J.M.B.); 2 Behavior and Perception group, Nestlé Research Centre, Lausanne 1000, Switzerland; nicolas.godinot@alimentarium.org (N.G.); Nathalie.Martin@rdls.nestle.com (N.M.); Ciaran_Forde@sics.a-star.edu.sg (C.G.F.); 3 Clinical Nutrition Research Centre, Singapore Institute for Clinical Sciences, Yong Loo Lin School of Medicine, National University of Singapore, Singapore 117599, Singapore

**Keywords:** oral processing behaviours, satiation, satiety, expected satiation, liking, appetite, nudge theory

## Abstract

Laboratory studies have demonstrated that experimental manipulations of oral processing can have a marked effect on energy intake. Here, we explored whether variations in oral processing across a range of unmodified everyday meals could affect post-meal fullness and meal size. In Study 1, female participants (*N* = 12) attended the laboratory over 20 lunchtime sessions to consume a 400-kcal portion of a different commercially available pre-packaged meal. Prior to consumption, expected satiation was assessed. During each meal, oral processing was characterised using: (i) video-recordings of the mouth and (ii) real-time measures of plate weight. Hunger and fullness ratings were elicited pre- and post-consumption, and for a further three hours. Foods that were eaten slowly had higher expected satiation and delivered more satiation and satiety. Building on these findings, in Study 2 we selected two meals (identical energy density) from Study 1 that were equally liked but maximised differences in oral processing. On separate days, male and female participants (*N* = 24) consumed a 400-kcal portion of either the “fast” or “slow” meal followed by an *ad libitum* meal (either the same food or a dessert). When continuing with the same food, participants consumed less of the slow meal. Further, differences in food intake during the *ad libitum* meal were not compensated at a subsequent snacking opportunity an hour later. Together, these findings suggest that variations in oral processing across a range of unmodified everyday meals can affect fullness after consuming a fixed portion and can also impact meal size. Modifying food form to encourage increased oral processing (albeit to a lesser extent than in experimental manipulations) might represent a viable target for food manufacturers to help to nudge consumers to manage their weight.

## 1. Introduction

Despite the well-documented health and economic consequences of obesity [1,2,3,4], incidence rates have been steadily increasing since 1980 [5]. This trend is generally attributed to a change in our dietary environment (e.g., [6,7,8,9]). Weight gain occurs when energy intake exceeds energy expenditure [10]. In response, individuals are advised to eat less and/or to exercise more. Despite the apparent simplicity of this advice, most attempts to reduce body weight are either unsuccessful or weight that has been lost is regained over time [11,12].

An alternative approach is to change the environment in which people live [13]. According to “nudge theory,” small targeted interventions (“nudges”) to change the environment (“choice architecture”) can have modest but cumulative effects on food intake and body weight [14]. A nudge should fulfil the following criteria: (i) it should target automatic processes; (ii) it should have a reliable effect on behaviour; (iii) it should not affect freedom of choice; and (iv) it should not significantly change economic incentives [14]. To date, studies evaluating nudge theory have explored the effects, inter alia, of reducing the availability of food [15] and of changing the position of a food on a menu [16]. In addition, reducing the portion size [17] and the energy density [18] of food can reduce both energy intake and body weight. A potential concern with the latter approach is that the pleasantness of a reformulated low energy-dense food can decrease over time (the “missing calorie effect”), which might undermine its efficacy as a weight-management product [19,20]. Here, we explored the extent to which it is possible to increase post-meal fullness and reduce meal size, simply by capitalizing on natural variation in the oral processing of everyday meals.

“Oral processing” refers to the pattern of behaviours associated with eating a food (e.g., bites, chews, swallows, and rate of eating). The degree to which foods require oral processing may be one aspect of our “obesogenic” environment that has changed in recent years [21]. Energy-dense foods are now available that can be consumed with relatively little oral processing [22]. In both humans [23,24,25] and rats [26], food texture (*i.e.*, viscosity, hardness, and chewiness) moderates the satiety response to food. For this reason, some researchers have suggested that liquid calories (*i.e.*, energy-containing drinks) deliver weaker satiation than equicaloric solid foods [27,28,29,30]. This is because oral exposure is limited [31]. Consistent with this interpretation, the effects of food viscosity on energy intake can be abolished when eating rate is controlled [23,32] and eating at a slower rate reduces meal size [33,34]. These acute effects are consistent with evidence that a faster eating rate is associated with a higher body mass index (BMI) [35,36,37,38]. Moreover, training obese adolescents to moderate their rate of eating produces a clinically significant and sustained reduction in body weight [39].

To date, studies have tended to explore effects of eating rate by instructing participants to consume a single food faster or slower, or by modifying the texture of food and assessing the effects of these manipulations on food intake and self-reported hunger [22,33,34]. Based on a recent meta-analysis [33], these types of experimental manipulations tend to achieve a 60% difference in eating rate (g/min; range: 17% to 143%). However, it remains to be determined whether the same variation in eating rate is observed across a range of unmodified everyday foods.

Previously, only one study has explored the association between eating rate and satiation across a broad range of commonly consumed products [40]. In this study, participants consumed taster portions (50 g) of a wide range of foods and beverages. Participants consumed these small portions (solid foods were pre-cut) and were instructed to eat without pausing between bites. Each participant was then offered *ad libitum* access to the same sample. Intake correlated positively with rate of consumption. However, drinks were included, together with a wide variety of foods (e.g., snacks, main meals, and confectionery). This raises questions about the extent to which this relationship would be observed across a range of otherwise similar meals that would normally be consumed in the same context. One other study has explored associations between oral processing behaviours and expected satiation [41]. Again, participants consumed small taster portions (50 g). Foods that were eaten at a slower rate, with smaller bite sizes, and with more orosensory exposure were expected to confer greater satiation. These findings are encouraging because they suggest that foods might be modified to increase satiation and satiety by promoting oral processing. However, to test this proposition further it is essential to demonstrate these relationships in full portions of everyday main meals, eaten on separate occasions. For the first time, this study quantified variation in oral processing across a range of unmodified main meals and explored the relationship between this variation and measures of satiation and satiety.

In Study 1, our primary objective was to establish the extent to which measures of expected satiation, satiation, and satiety are associated with differences in the oral processing of commonly consumed main meals. Female participants attended the laboratory over 20 lunchtime sessions. In each session, they consumed a 400-kcal portion of a different commercially-available pre-packaged main meal. Prior to consumption, expected satiation was assessed. During each meal, oral processing behaviours were characterised using video-recordings of the mouth and real-time measures of plate weight. Hunger and fullness ratings were elicited pre- and post-consumption. In Study 2, we sought to; (i) demonstrate that differences in oral processing (observed in Study 1) are sufficient to influence food intake and; (ii) establish that the effects of oral processing can be observed when both males and females are included in the sample and across different test foods that are matched for their portion size (g). We selected two meals (same energy density) from Study 1 that had very different effects on oral processing but were equally liked. On separate days, participants consumed a 400-kcal portion of either the “fast” or “slow” meal, followed by an *ad libitum* meal.

## 2. Study 1

### 2.1. Materials and Methods

#### 2.1.1. Participants

In similar studies where participants have consumed many different foods, the number of participants sampling each food was between 3 and 13 [40,42]. For this study, 12 participants were recruited from the population of Bristol (UK) via an online database of volunteers belonging to the Nutrition and Behaviour Unit (NBU). These volunteers had previously joined the database following advertisements posted within the University and the wider community (e.g., in local newspapers) for participants to take part in eating behaviour research studies. At the time of conducting this study, 5905 volunteers (58% female) were registered on the database. Volunteers comprised University students, staff and members of the public, and a wide range of ages was represented (18–83 years). Since there are known gender differences in the amount of food that is required to achieve satiation [43], only females were tested. Participants were told that the purpose of the study was to investigate the acceptability of commercially available foods. Their mean age was 23.3 years (S.D. = 10.0; range = 19–55) and their mean BMI was 23.0 kg/m^2^ (S.D. = 3.3; range = 19.2–29.7). Respectively, their mean scores for Dutch Eating Behaviour Questionnaire (DEBQ) restraint, emotional eating and externality were 2.3 (S.D. = 0.4; range = 1.4–2.7), 2.5 (S.D. = 0.6; range = 1.5–3.9) and 3.5 (S.D. = 0.5; range = 2.5–4.5). Participants were excluded if they were; (i) vegetarian or vegan, (ii) not fluent in English, (iii) trying to lose weight, (iv) taking medication (with the exception of oral contraceptive pills), or (v) smoking more than five cigarettes a day. Anyone reporting a food allergy or intolerance was also excluded. In remuneration for their assistance, all participants were offered £50 (Sterling) upon completion of the study. For both Studys 1 and 2, ethical approval was granted by the University of Bristol Faculty of Science Human Research Ethics Committee (Study 1 approval code = 251012599, Study 2 approval code = 2609133982). Written consent was obtained from all participants including specific permission to store video footage on our secure server.

#### 2.1.2. Test Meals

Twenty commercially available pre-packaged meals (supplied by Sainsbury’s Supermarkets Limited, Holborn, London, UK) were selected for inclusion in the study. These were chosen to represent a range of commonly consumed foods in the United Kingdom (UK). Equal-caloric portions of each meal were served during each test session. Sandwiches are commonly consumed for lunch in the UK. We examined the energy content of 495 commercially available sandwiches [44] and found an average energy content of 393 kcal. To reflect this typical meal size, we served 400-kcal portions of the test foods (see Figure S1 for images of the test meals, as served). Each test meal was cooked according to the manufacturer’s recommendations and the total cooked weight (without packaging) was used to calculate; (i) the weight for a 400-kcal portion and (ii) the correct proportion (%) of each component (e.g., fish, chips, peas) to serve in multi-component meals. See Table 1 for associated portion sizes and the macronutrient composition of each test meal.

After testing three participants, one of the pre-packaged meals (chicken and prawn paella) was withdrawn by the manufacturer and replaced by another version of a slightly lower energy density. A new portion size for this version was calculated and the revised weight was served to participants. Both versions are shown in Table 1 and Figure S1. The serve weight and temperature of each meal was recorded as it left the kitchen.

Before consuming each test food, participants completed an assessment of liking and expected satiation (see the measures section below). To limit cooling during this period, the test foods were presented on a pre-heated 255-mm diameter white plate. Participants were offered 250-mL of water with each test meal. No additional water was provided. Across participants, the test foods were presented in a different random order. Participants were not told in advance which meal they would be sampling during each session.

#### 2.1.3. Measures

##### Liking

In each session, the participants were instructed to taste a single mouthful of the test meal. For multi-component meals, participants were asked to ensure that they had tasted a small amount of each component of their meal. Liking was assessed using a 100-mm visual analogue scale (VAS) rating (Heading: “How much do you like the taste of this food?”; anchor points: “Not at all” and “Extremely”).

##### Expected Satiation

A “method of constant stimuli” was used to quantify the expected satiation of the test meals. For a detailed account of this methodology the reader is referred to Brunstrom, Shakeshaft and Scott-Samuel [45]. Participants were given a 400-kcal portion of the test meal on a white 255-mm diameter plate. A photograph of a “comparison” food (actual displayed size: 750 pixels × 750 pixels) was presented on a Samsung SyncMaster XL2270HD widescreen TFT-LCD monitor (screen resolution: 1920 × 1080 pixels). Participants were instructed to “Look at the portion of the food in front of you. Imagine you are going to eat this portion of food RIGHT NOW. Now look at the portion of food on the screen. Imagine you are going to eat this portion of food INSTEAD. Decide which food would leave you feeling the most full (immediately after consumption).” Responses were made by depressing the up and down arrow-keys to indicate whether the comparison food (on screen) or the test food (in front of them) was expected to be more filling.

The two comparison foods were chicken tikka masala with rice (Indian range, Sainsbury’s Supermarkets Ltd. (UK); Nutritional information per 100 g—175 kcal, 7.4 g protein, 19.4 g carbohydrate, 7.6 g fat, 4.5 g fibre) and spaghetti bolognese (Italian range, Sainsbury’s Supermarkets Ltd.; Nutritional information per 100 g—141 kcal, 7.2 g protein, 16.2 g carbohydrate, 5.3 g fat, 2.1 g fibre). As in previous studies [45,46,47,48], our choice of comparison foods was motivated by a concern to present stimuli that are highly familiar. Chicken tikka masala with rice and spaghetti bolognese were selected based on familiarity data obtained from a similar population [19]. Across trials, each comparison food was presented alternately and 56 times (112 trials in total). The size of the comparison food changed across trials and was selected from a set of images depicting portions ranging from 20 to 1000 kcal, in 20-kcal steps. Particular care was taken to maintain a constant lighting condition and viewing angle in each image.

In each trial, the portion size of the comparison food was determined using an adaptive probit estimation algorithm (APE) [49]. Again, for more information the reader is referred to Brunstrom *et al.* [45]. Briefly, based on an assessment of previous responses, this algorithm selects comparison values that are likely to be centred on a probable match between the test food and the comparison (point of subjective equality, PSE). Subjectively, this means that decisions tend to become more difficult with increasing trial number. The PSE represents the point at which the test food is likely to be selected 50% of the time. This value is important because it indicates the amount of the comparison (in kcal) that is expected to be equally as filling as the test meal. The APE routine and the code for presenting the stimuli were written in Matlab (Version R2013a; Mathworks, Natick, MA, USA). The graphical interface was implemented using Cogent Graphics (developed by John Romaya at the Laboratory of Neurobiology at the Wellcome Department of Imaging Neuroscience, UK). After completing half of the trials, the participants were offered an opportunity to take a break. This task took approximately five minutes to complete.

For each participant and for each test meal, a pair of PSE values was recorded (PSE^1^ = spaghetti bolognese and PSE^2^ = chicken tikka masala and rice). A measure of expected satiation was derived from their geometric mean.

##### Oral Processing Behaviours

Oral processing was assessed using a combination of real-time measures of plate weight and video-recordings of the mouth. These measures are described in turn below.

Real-Time Measures of Plate Weight Across a Meal: Following previous studies [50,51,52,53], initial eating rate (g/s) and change in eating rate (acceleration/deceleration; g/s^2^) were assessed using a continuous measure of plate weight during each meal. An Ohaus Defender™ 3000 series scale (maximum weight: 10 kg, sensitivity: ±1 g) was connected to a PC using an RS232 interface and placed on a table in front of the participant. During the meal, custom software recorded the weight of the plate every second to the nearest gram. Participants were unable to see the readout from the balance.

To process the continuous meal-weight data, a script was developed in Matlab (version R2013a; Mathworks, Natick, MA, USA; detailed in Text S1). First, we removed any data points that had been recorded before the meal began or after it had terminated. Second, the raw data were filtered to remove noise caused by events such as the application of cutlery pressure to the plate. In the first instance we filtered the data by calculating a median value at every second based on a roving three-second window around each time point. The median values at adjacent time points were then compared in an iterative loop. If the median weight recorded at the second time point (*t*2) was greater than at the preceding time point (*t*1), the value at *t*2 was replaced with the value at *t*1. In addition, to counteract any instances where participants lifted the plate momentarily off the scale, any decrease in weight that was greater than 25 g was replaced with the value that preceded it. Our decision to use a 25-g threshold was based on previous studies [41,54]. These have reported typical bite sizes between 5 and 8 g across participants and across foods, suggesting that a 25 g decrease is unlikely to be attributed to a genuine eating event. Finally, the filtered weight-loss data were then inverted to generate cumulative intake data. These intake data were normalised by subtracting the first signal value from the entire time series before a cumulative intake curve (CIC) was fitted. Consistent with previous methods for characterising eating behaviour [51,55], a CIC was plotted by fitting a quadratic equation to the cumulative intake data using the formula *y = ax^2^ + bx + c*. In this equation, *y* = amount of food eaten, *x* = time, *a* = change in the slope of the curve over time (acceleration or deceleration rate), *b* = initial eating rate, and *c* = food intake at the start of the meal (*i.e.*, this value should be very close to 0). For each participant, this process yielded an estimate of initial eating rate (g/s) and change in eating rate (acceleration/deceleration; g/s^2^), for each of the 20 test meals. Respectively, acceleration and deceleration across a meal is reflected in positive and negative slopes for *a*. We observed an excellent fit to all CICs (average *R*^2^ = 0.99; range = 0.96–1.00).

Real-Time Video Recordings: Following previous studies [25,41], oral processing behaviours were also assessed using a video recording of the mouth. A Logitech C270 webcam was positioned at the top of the monitor, approximately 0.8 m from the participant. Before taking part, the participants were aware that they would be recorded during each session while eating. However, particular care was taken to minimise any disruption during the meal. Specifically, the recording light on the webcam was disabled and participants were unable to see an image of themselves during filming. Informal analysis of the videos suggested that participants paid little attention to the webcam.

To standardise the extraction of quantitative data from the 240 raw video recordings, a customised coding scheme was developed (available on request) using ANVIL 5.0 video annotation software [56]. For each video, an experimenter coded the start (defined as the first bite) and end (defined as the final swallow) of the meal and recorded each individual bite, chew and swallow. This enabled us to automatically demarcate specific periods of orosensory exposure (a period during which food was in the mouth) from inter-bite intervals (a period between a swallow and a subsequent bite with no food in the mouth).

Coding of the 240 videos was divided between three researchers. To establish inter-rater reliability, all researchers coded a set of videos from a single participant (20 videos—8.3% of the total videos). Across the three coders, intra-class correlations coefficients (ICC; two-way mixed-effects absolute agreement) were calculated for total number of bites, total number of chews, total number of swallows, total orosensory exposure time (s), total inter-bite interval (s), and meal duration (s). The average-measures ICC for each of these variables was greater than 92%. For this participant, analysis of each oral processing measure was based on an average value computed across the three coders.

After the videos had been coded, we quantified oral processing behaviours that have been shown to affect satiation previously; (i) eating rate [57]; (ii) total orosensory exposure time [58]; (iii) total inter-bite interval [59]; (iv) average bite size [60,61]; (v) chews per mouthful [62,63]; and (vi) bite rate [64]. Eating rate (kcal/s) was calculated by dividing the number of calories served (400 kcal) by the total meal duration (s). Respectively, total orosensory exposure time (s) and total inter-bite interval (s) were computed by summing the durations of orosensory exposure and inter-bite intervals across the meal. Average bite size (g) was calculated by dividing the weight of food by the total number of bites. Chews-per-mouthful was calculated by dividing the total number of chews by the total number of bites. Finally, bite rate (number of bites per s) was calculated by dividing the total number of bites by the total meal duration (s).

##### Satiation and Satiety

Participants rated their hunger (Heading: “How hungry do you feel right now?”; anchor points: “Not at all hungry” and “Extremely hungry") and fullness (Heading: “How full do you feel right now?”; anchor points: "Not at all full” and “Extremely full”) on a 100-mm paper and pencil VAS. Based on previous observations that hunger and fullness ratings are highly correlated [65] and to limit the number of statistical tests performed, from each pair of values, a composite “fullness score” was calculated using the formula ((100 − hunger) + fullness)/2). We note that an identical pattern of data were obtained when hunger and fullness ratings were analysed separately. Ratings were taken at the beginning of each session and immediately after eating. Participants were also given further hunger and fullness ratings to complete one hour, two hours, and three hours after eating. To assess satiation, we analysed the composite fullness score obtained immediately after eating (0 min). To generate a single “satiety score”, for each participant and for each test meal, the trapezoidal rule was applied to calculate the total area under the curve (AUC) using the 0, 60, 120 and 180 min composite fullness scores.

#### 2.1.4. Procedure

Each participant attended the NBU at the same time of day every weekday for twenty test sessions. Sessions could be scheduled between 12:00 and 14:00. To standardise satiety, participants were instructed to consume their normal breakfast and to abstain from eating and consuming calorie-containing beverages for at least three hours prior to arrival. To promote compliance, on arrival, participants were asked to report the amount of time since they last ate or consumed a calorie-containing beverage. Baseline appetite (hunger and fullness) was assessed before each test meal. Participants were then instructed to taste a single mouthful of the meal before completing measures of liking and expected satiation. Video and weight recordings were initiated simultaneously and the participants were then instructed to eat the entire meal at their usual pace. Participants were reminded to neither move the plate nor place cutlery on it. Participants ate alone. Immediately after meal completion, the participants completed another set of appetite ratings. They were then instructed to abstain from eating and from consuming calorie-containing beverages for three hours. After one, two and three hours they re-rated their appetite. To remind the participants to provide these ratings they were issued a pre-programmed electronic timer [66,67].

At the end of the final session participants completed the DEBQ [68]. We also assessed the extent to which our participants were aware of the aims of the study. Specifically, they were asked to provide a written response to the question, “In your opinion, what was the purpose of this study”? Finally, weight, height and age were recorded. Debriefing took place by email, after all of the data had been collected.

#### 2.1.5. Data Analysis

All data were analysed using IBM SPSS Statistics 21 (Armonk, NY, USA). Critical *p*-values < 0.05 were considered significant. One participant did not consume the entire portion of one of her test meals (sausage and mashed potato). Data for this session were excluded and treated as missing. The remaining 239 sessions were included in our analyses. Due to experimenter error, food temperature was not recorded in one session (beef lasagne).

In the first instance, we assessed differences in the time since last eating, baseline fullness, and food temperature, across meal type. For each of these variables, we ran a repeated-measures ANOVA with “meal type” (20 levels) as a within-subjects factor. Across meals, we found no significant difference in food temperature or in the time since last eating. However, baseline fullness did differ significantly (*p* = 0.04) (see Table 2 for means and associated statistics). The order of meal presentation was randomised across participants and at this point in the protocol the participants had not seen their test meal. Therefore, we suspect this effect of meal type is a chance finding. Nevertheless, baseline fullness was included as a controlling factor in the calculation of partial correlations between variables of interest. From the outset we sought to demonstrate that variation exists in the oral processing of, and satiety responses to, commonly consumed pre-packaged meals in the UK. Accordingly, we used repeated-measures ANOVAs (meal type as a within-subjects factor) to explore across-meal differences in measures of oral processing, expected satiation, and rated fullness (after eating the fixed portion).

Our primary concern was to determine whether specific aspects of oral processing are reliably associated with expected satiation, satiation and satiety, across test meals. Therefore, we pooled data across participants and calculated mean values for each food and for each measure. Pearson’s partial correlation coefficients (controlling for baseline fullness) were calculated to explore the relationship between each measure across foods (this strategy is referred to here as an “across-food analysis”). All partial correlations are reported according to widely accepted recommendations regarding effect sizes [69]. Respectively, *r* values of 0.10, 0.30 and 0.50 are interpreted as a small, medium and large effect. The same analysis strategy was used to analyse; (i) inter-relationships between our measures of oral processing; (ii) the relationship between liking and oral processing; and (iii) the relationships between expected satiation, satiation, and satiety.

A potential concern is that any observed relationships might be mediated by differences in the weight of the test meals. Foods that are larger will take longer to eat. Therefore, they will require greater oral processing (chews/swallows/bites, *etc.*). In addition, it is well established that satiation is influenced by food volume [70], potentially via gastric distension [71]. To explore the independent effects of oral processing behaviours on satiation, we selected two subsamples of the 20 foods that were closely matched for their energy density (and therefore food weight). See Table 1 for a list of these foods and their associated subgroup. The “low energy-dense” subgroup (1.3 kcal/g) comprised five meals. Their largest difference in weight was 20.7 g. The “high energy-dense” subgroup (1.8 kcal/g) also comprised five meals. The largest difference in the weight of these meals was 7.3 g. In each subgroup, we repeated our across-foods correlational analyses for the relationships between oral processing and our variables of interest (expected satiation, satiation and satiety). In so doing, we were able to explore predictors of satiation in foods that are matched for their size. This strategy is referred to as a “high- and low- energy-density subgroup analysis”.

### 2.2. Results

#### 2.2.1. Across-Meal Variability

Table 2 summarises the mean (±S.D.) scores for each of our measures and for each test meal. Respectively, on a calorie-for-calorie basis, the percent difference between the largest value and the smallest score for expected satiation (kcal), liking (mm), fullness after a fixed portion (mm) and satiety (fullness AUC) was 93%, 61%, 33% and 32%. We also observed substantial variation in all of our measures of oral processing. For example, eating rate varied from 0.9 kcal/s for vegetable biryani to 2.0 kcal/s for beef stew with dumplings. In addition, orosensory exposure time differed by 87% (169.0 s for sausage and mashed potato *versus* 426.9 s for vegetable biryani). In all cases, these values are reflected in large and significant F ratios associated with across-food differences (see Table 2).

#### 2.2.2. Inter-Relationships between Oral Processing Behaviours

Table 3 shows the inter-relationships between our various measures of oral processing. A faster eating rate (kcal/s) was associated with less orosensory exposure time (s) (*r* = −0.92, *p* < 0.001) and with shorter inter-bite intervals (s) (*r* = −0.65, *p* = 0.003). A faster initial eating rate (g/s) was associated with fewer chews per mouthful (*r* = −0.73, *p* < 0.001), a larger bite size (g) (*r* = 0.77, *p* < 0.001) and deceleration during a meal (g/s^2^) (*r* = −0.74, *p* < 0.001). Average bite size (g) was negatively correlated with orosensory exposure time (s) (*r* = −0.47, *p* = 0.04) and with the number of chews per mouthful (*r* = −0.52, *p* = 0.02), and chews per mouthful was associated with a lower bite rate per second (*r* = −0.68, *p* = 0.001). No other relationships reached significance (all *p* > 0.05).

#### 2.2.3. Relationships between Oral Processing, Satiation and Satiety

**Figure 1:**
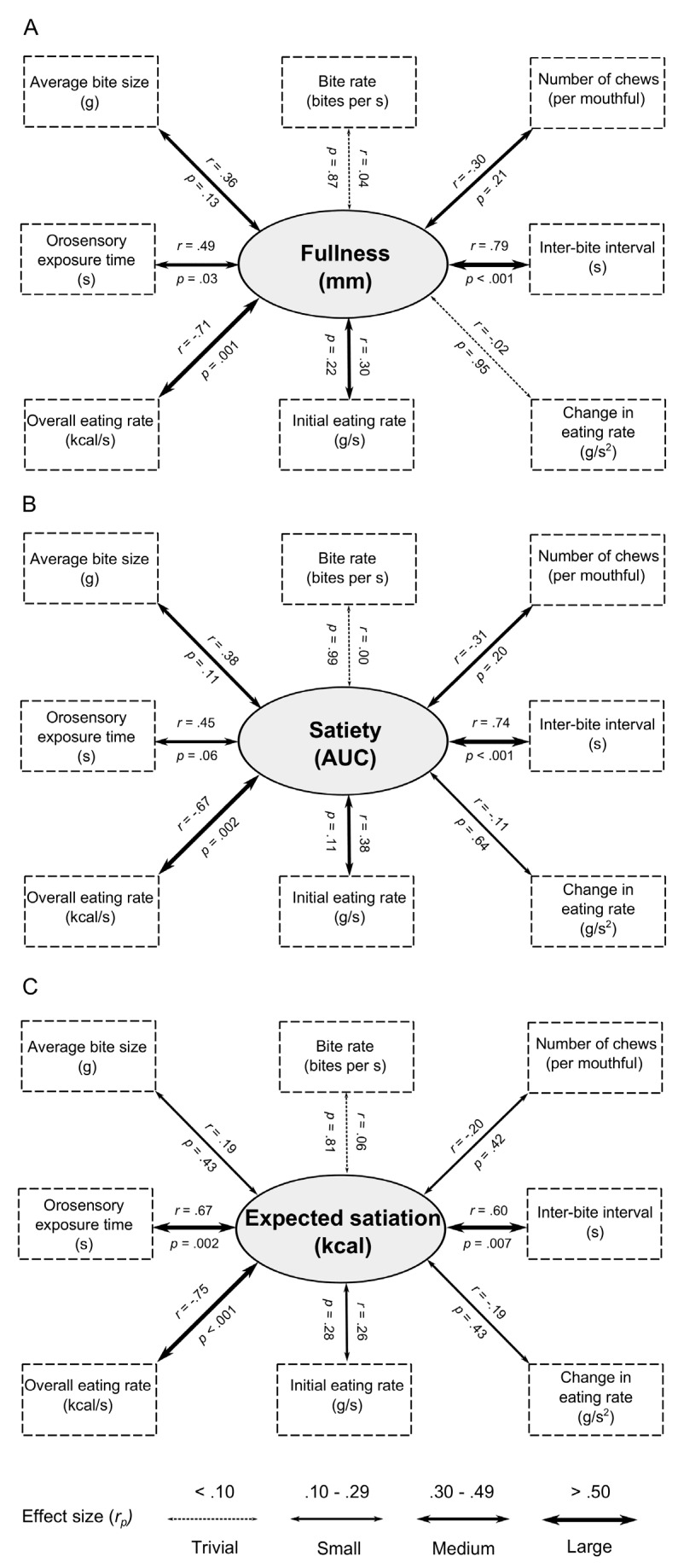
Partial correlations (*r_p_*, controlling for baseline composite fullness) between oral processing behaviours and fullness after consuming the fixed portion (satiation—panel (**A**)); fullness for three hours after eating (satiety—panel (**B**)); and expected satiation (panel (**C**)) Effect sizes are denoted by the width of each arrow.

Across foods analysis: Figure 1 summarises the relationships between each of the oral processing measures and fullness immediately after eating (see panel A), and with satiety scores (panel B). The same pattern of results was observed in both sets of analyses. Foods that were eaten at a slower rate (kcal/s) were associated with greater post-meal fullness (*r* = −0.71, *p* = 0.001) and with greater satiety (*r* = −0.67, *p* = 0.002). In addition, foods with greater orosensory exposure time (s) were associated with greater post-meal fullness (*r* = 0.49, *p* = 0.03) and with greater satiety (*r* = 0.45, *p* = 0.06 —a marginal result). Foods with a longer inter-bite interval (s) were also associated with greater satiation (*r* = 0.79, *p* < 0.001) and with satiety (*r* = 0.74, *p* < 0.001).

High and low energy-density subgroup analyses: Table 4 summarises the relationships between oral processing behaviours and fullness immediately after eating, and with satiety scores. Values are provided for foods in the low and high energy-density subgroups, separately. In the low energy-density subgroup, participants experienced greater satiety after consuming foods with more chews per mouthful (*r* = 0.94, *p* = 0.056—a marginal result), a longer orosensory exposure time (s) (*r* = 0.96, *p = 0.*04) and where they showed deceleration (g/s^2^) (*r* = −0.98, *p* = 0.02). In the high energy-density subgroup, participants experienced greater satiety after consuming foods where they showed less deceleration (*r* = 0.99, *p* = 0.009). In addition, despite the fact that we reduced our power from 20 foods to 5 within each subgroup, we note that the following variables demonstrated moderate to large effect size relationships with satiation and satiety; eating rate (kcal/s), inter-bite interval (s) and average bite size (g).

#### 2.2.4. Relationships between Oral Processing and Expected Satiation

Across foods analysis: Figure 1 (panel C) summarises the relationships between expected satiation and oral processing. Foods that were eaten at a slower rate (kcal/s) (*r* = −0.75, *p* < 0.001) were expected to deliver more fullness. In addition, foods with a longer orosensory exposure time (*r* = 0.67, *p* = 0.002) and inter-bite interval (*r* = 0.60, *p* = 0.007) were expected to be more filling. The associations between expected satiation and initial eating rate (g/s), deceleration rate (g/s^2^), average bite size (g), chews per mouthful and bite rate (bites/s) were all non-significant and reflected a trivial or small effect size (all *r* < 0.30).

High and low energy density subgroup analyses: Table 4 summarises the relationships between expected satiation and oral processing in the low and high energy-density subgroups, separately. In the low energy-density subgroup many of the relationships that we observed in our across-food analysis were preserved. Specifically, the relationships between expected satiation and eating rate (kcal/second) (*r* = −0.99, *p* = 0.002) and orosensory exposure time (s) (*r* = 0.97, *p* = 0.03) were preserved, even in foods matched for their weight. This was the case despite the fact that we reduced our power from 20 foods to 5. In both subgroups, expected satiation was related to the following variables with a moderate to large effect-size; eating rate (kcal/s), initial eating rate (g/s), orosensory exposure time (s), average bite size (g), and number of chews per mouthful.

#### 2.2.5. Relationships between Oral Processing and Liking

**Figure 2:**
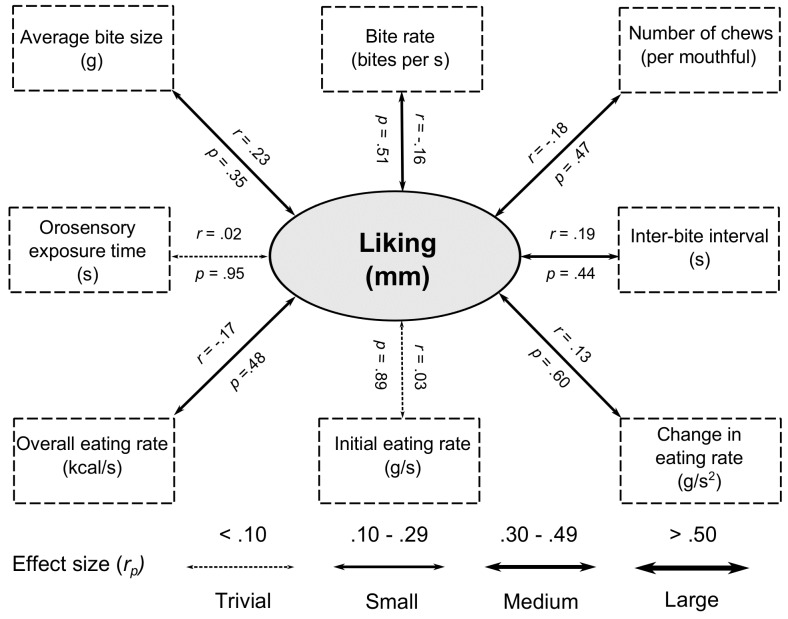
Partial correlations (*r_p_*, controlling for baseline composite fullness) between oral processing behaviours and food liking (mm). Effect sizes are denoted by the width of each arrow.

There were no significant correlations between liking and any of the oral processing measures (all *p >* 0.35; see Figure 2). All associations had a small or trivial effect size (all *r < 0.*23).

#### 2.2.6. Relationships between Expected Satiation, Satiation and Satiety

Foods that were expected to deliver greater satiation (kcal) were associated with greater post-meal fullness (*r* = 0.78, *p* < 0.001) and with greater satiety (*r* = 0.77, *p < 0*.001).

#### 2.2.7. Demand Awareness

In response to the open-ended question about the purpose of the study, 33.3% thought that the study was assessing liking and fullness after consuming different supermarket pre-packaged meals. A smaller proportion (25%) mentioned speed of eating but did not connect this with fullness. Instead, they suggested that we were assessing the effects of liking on speed of eating. Other participants offered alternative suggestions. None related to the objectives of the study.

## 3. Interim Discussion

In Study 1 we sought to quantify variation in the oral processing of pre-packaged meals and to determine whether this is associated with differences in post-meal fullness and expected satiation. In the first instance, we wanted to establish whether there might be meaningful differences in oral processing across a single product category—commercially available pre-packaged meals. Our analysis revealed large differences in every measure of oral processing. Forde *et al.* [41] found that sample portions (50 g) of savoury meal components had an orosensory exposure time that ranged between 28.0 s for canned tomatoes to 349.0 s for tortilla chips (a 12 fold difference). In our study, orosensory exposure varied from 169.0 s for a 285 g portion of sausage and mashed potato to 426.9 s for a 316 g portion of vegetable biryani (a 2.5 fold difference). Nevertheless, we still observed strong relationships between our measures of oral processing. Consistent with related observations [31], we found a very strong negative relationship between eating rate (kcal/s) and orosensory exposure time (*r* = −0.92), and between eating rate and inter-bite interval (*r* = −0.65).

We also observed large variation in the other dependent measures. Respectively, liking, expected satiation, post-meal fullness, and actual satiety varied across our test foods by 61%, 93%, 33%, and 32%. This is consistent with previous observations that equal-energetic servings of different foods differ greatly in their expected [72] and actual [42] satiation and satiety.

We hypothesised that our measures of oral processing would correlate with measures of satiation and satiety. Our across-food analysis revealed strong relationships between satiation (fullness) and more orosensory exposure time, longer pauses between bites, and a slower eating rate. These measures of oral processing also predicted satiety (fullness post-meal over three hours). Together, these findings are consistent with studies that have manipulated oral processing by modifying food texture or by explicitly instructing participants to eat faster or slower than normal [21,24,25,33,34,60]. However, this is the first study to demonstrate that these relationships can be observed in the variation of oral processing associated with a range of unmodified main meals.

Here, we have taken a correspondence between oral processing, post-meal fullness and satiety to indicate evidence for a causal effect of oral processing on satiation and satiety. However, we acknowledge that alternative explanations exist. One possibility is that differences in food volume mediate these relationships (*i.e.*, items that are larger require more oral processing and also promote greater fullness). To address this concern, in our subgroup analyses, we selected two subsamples of the 20 foods that were closely matched for their energy density. Despite the fact that we reduced our power from 20 to 5 foods, a significant relationship was still observed between orosensory exposure time and satiety (AUC response). Although, we note that this was only evident in our low energy-density subsample. In our assessment of post-meal fullness, correlations failed to reach significance. Therefore, based on the current data, we are unable to dismiss an explanation based on a possible mediating effect of food volume. Nevertheless, in both subsamples, the effect sizes were generally unmodified, suggesting a lack of statistical power (caused by a reduction in sample size) rather than a change in trend. For example, and importantly, in both the high and low energy-density sub-groups, moderate to large effect size negative correlations were still observed between eating rate, satiation and satiety. Explanations based on the small differences in food volume cannot account for this pattern of results.

In our across-food analysis of the relationships between oral processing behaviours and expected satiation, we also observed the same significant associations with orosensory exposure time, inter-bite interval and eating rate. These data suggest that the effects of oral processing on satiation and satiety might be learned and anticipated before a meal begins. This relationship with expected satiation is important, because expected satiation plays a central role in the control of meal size and food intake [48,73]. By contrast, we failed to find significant relationships between liking and any oral processing characteristic. All of the associated effect sizes were small or trivial, suggesting that variations in liking of everyday meals is not sufficient to impact oral processing.

We also observed that foods that were expected to deliver greater satiation before eating were associated with greater post-meal satiation and satiety. These associations are consistent with previous research which has manipulated expected satiety for a fruit smoothie and shown that this has a marked effect on post-meal hunger and fullness [67,74].

Together, the results suggest that foods that are eaten slower, with longer pauses between bites, and more orosensory exposure, deliver more satiation/satiety and that these relationships are expressed in beliefs associated with the expected satiation of foods. However, three outstanding questions remain. First, we have established relationships between oral processing and subjective ratings of hunger and fullness (fullness composite score). Although these measures may reflect a proclivity to engage in eating they may not predict the amount of food that will be eaten [73,75,76]. It remains to be determined whether the variation in oral processing observed here has a meaningful effect on subsequent food intake.

Second, as noted earlier, our subgroup analysis involved a comparison of foods that were closely matched for their weight. Our strategy reflects an attempt to control for the effect of differences in food volume (weight) to show independent effects of oral processing. Even after controlling for food weight, several relationships between oral processing, expected satiation and actual satiety remained and the effects sizes of the relationships were generally unmodified. However, as noted earlier, our subgroup analysis also suffered from reduced statistical power. To address this concern, the effects of oral processing should be explored in foods that are matched for their energy density. Finally, in Study 1, we tested only females. It remains to be determined whether relationships with oral processing are also observed in males.

In response to these issues we conducted a second study. Specifically, we had two objectives. First, we explored whether the differences in oral processing that were observed in Study 1 can influence food intake at a subsequent *ad libitum* meal. Second, we sought to establish whether the effects of oral processing can be observed when both males and females are included in the sample and across different test foods that are otherwise matched for their portion size (g) and energy density.

## 4. Study 2

### 4.1. Materials and Methods

#### 4.1.1. Overview

Participants attended the NBU over four sessions. In each session they were presented with and consumed a fixed portion (400 kcal) of beef stew with dumplings (the fast meal) or fish, chips and peas (the slow meal). Participants were then offered either the same food or a dessert. Specifically, participants rated their fullness and were presented with an *ad libitum* portion of either the same food as the previous fixed portion or a lemon mousse. This manipulation enabled us to explore the extent to which oral processing of a fixed portion differentially impacts subsequent *ad libitum* intake, when presented with more of the same food or a dessert. One hour later, we gave participants *ad libitum* access to snack foods (tortilla chips and cookies). We hypothesised that participants would consume less food after the slow meal and that this difference in energy intake would not be compensated during the subsequent snack meal.

#### 4.1.2. Test Meals

In the first instance, we identified two test meals from Study 1 that; (i) maximised differences in oral processing; (ii) were matched for food weight (energy density); and (iii) were equally liked. In both the high and the low energy-dense subgroups separately, for each oral processing measure, we calculated the absolute value of the standardised mean difference (SMD) between each food pair. These SMDs were summed to determine the food pair that generated the largest difference in oral processing. Table S1 summarises these SMDs across all food pairs. The largest total SMD was observed between beef stew with dumplings and fish, chips and peas (see Table 1 for the portion size and macronutrient information of these fixed portion meals). In addition, a paired-samples *t*-test revealed that there was no significant difference in liking between these meals in Study 1 (*t*(11) = −1.03, *p* = 0.33). The weight of these meals differed by only 3.2 g.

Immediately after participants had consumed the fixed portion, an *ad libitum* meal (1200 kcal) of either the same food (“same food condition”) or a lemon mousse (“dessert condition”) was served. In the same food conditions, participants were served either 668.8 g of fish, chips and peas (Nutritional information per serving—37.5 g protein, 150.0 g carbohydrate, 45.0 g fat, 20.6 g fibre) or 659.2 g of beef stew with dumplings (Nutritional information per serving—58.4 g protein, 101.8 g carbohydrate, 52.1 g fat, 43.4 g fibre). In the dessert conditions, participants were served 652.2 g of lemon mousse (Nutritional information per serving—18.3 g protein, 157.2 g carbohydrate, 54.8 g fat, 3.3 g fibre) One hour later, an *ad libitum* snack meal (1000 kcal) was provided. This comprised 500 kcal of tortilla chips (102.3 g; Nutritional information per serving—6.3 g protein, 63.7 g carbohydrate, 24.4 g fat, 4.0 g fibre) and 500 kcal of chocolate chip cookies (100.0 g; Nutritional information per serving—5.9 g protein, 64.3 g carbohydrate, 23.9 g fat, 2.0 g fibre). All foods were supplied by Sainsbury’s Supermarkets Limited, Holborn, London. Fish, chips and peas and beef stew with dumplings were always served on a 255-mm diameter white plate. Lemon mousse was served in a 1.5-L clear glass bowl and tortilla chips and cookies were each served in separate 1.0-L clear glass bowls. Participants were offered a 250-mL glass of water with every meal. No other water was made available.

#### 4.1.3. Participants

To calculate the required sample size, we performed a power calculation on the change in fullness (from baseline) after eating the fast and the slow food in Study 1. A paired-samples *t*-test revealed a significantly greater increase in fullness after consuming the fish, chips and peas (*t*(11) = −2.98, *p* = 0.01). On this basis, we estimated that 13 participants would be required to detect differences in satiation with 80% power and α = 0.05. To fully counterbalance the order of conditions, we recruited 24 participants (12 females, 12 males) from our volunteer database. The mean age for male participants was 21.2 years (S.D. = 2.7; range = 19–29) and their BMI was 22.7 kg/m^2^ (S.D. = 2.8; range = 19.3–28.6). Respectively, mean scores for DEBQ restraint, emotional eating and externality in males were 2.2 (S.D. = 0.8; range = 1.2–3.4), 2.3 (S.D. = 0.8; range = 1.0–3.9) and 3.2 (S.D. = 0.9; range = 1.3–4.8). The mean age for female participants was 24.3 years (S.D. = 4.7; range = 20–33) and their BMI was 20.8 kg/m^2^ (S.D. = 2.3; range = 18.0–26.0). Respectively, mean scores for DEBQ restraint, emotional eating and externality in females were 2.3 (S.D. = 0.6; range = 1.0–3.3), 2.3 (S.D. = 0.8; range = 1.1–3.4) and 3.4 (S.D. = 0.5; range = 2.4–4.2). Participants were informed that the study was investigating the acceptability of commercial foods. The exclusion criteria were the same as in Study 1. In addition, we excluded participants who had assisted with Study 1. All were offered £40 (Sterling) in remuneration for their assistance.

#### 4.1.4. Measures

##### Sensory Ratings

To assess whether participants were aware of any textural difference between the fast and slow meal, we included a number of sensory ratings that were completed after tasting each food. These were based on previous published research [25] and included; (i) flavour intensity; (ii) sweetness; (iii) saltiness; (iv) savouriness; (v) firmness; and (vi) chewiness. Participants were asked “How (INTENSE is the FLAVOUR of/SWEET is/SALTY is/SAVOURY is/FIRM is/CHEWY is) this food”? (anchor points: “Not at all” and “Extremely”).

##### Oral Processing Behaviours

We introduced two minor changes to our procedure for assessing oral processing. First, real-time recording of plate weight was achieved covertly by embedding the balance in a table (80 cm wide × 80 cm deep × 70 cm high). The table was covered with a tablecloth and a placemat was positioned to demarcate the location of the scale. Second, in Study 1 we noticed that some participants took more than one bite from the same forkful (“fork cleaning” or “knife cleaning”). Therefore, in Study 2, we modified our video coding scheme to separate fork-cleaning bites from bites where food was removed from the plate.

Video coding was implemented for the 96 videos of the fixed portion meals. (Four meals × 24 participants—the four meals comprised a pair of slow meals and a pair of fast meals. In each pair, one meal was followed by a dessert and the other by the same meal). Coding was divided between two researchers. To assess inter-rater reliability, three out of the 24 participants were selected at random and all of their fixed portion videos were coded by both researchers (12 videos—12.5% of the total videos). Following Study 1, ICCs (two-way mixed-effects absolute agreement) were calculated for total number of bites, total number of chews, total number of swallows, total orosensory exposure time (s), total inter-bite interval (s), and meal duration (s). None of the average measures ICCs was less than 93%. For these three participants, analysis of each oral processing measure was based on an average value computed across the two coders.

Some participants did not consume any of their *ad libitum* meal (*n =* 7, 5 of these cases occurred in dessert conditions). In these cases, for the real-time measures of plate weight, their coefficients were entered as missing. In general, we observed an excellent fit to most CICs, both for the fixed portion (average *R^2^* = 0.97; range = 0.41–1.00) and the *ad libitum* meals (average *R^2^* = 0.91; range = 0.06–1.00). However, we were concerned about the fits of a few of our models. Inspection of the CIC plots revealed that some participants rested on the balance during the meal (this was particularly the case for lemon mousse). To address this, we excluded and treated as missing all CICs where the *R^2^* value was lower than 0.80 (80% of the variance explained by our model). For the fixed portion meals, this resulted in the exclusion of 5 out of 96 meals (revised average *R^2^* = 0.98; revised range = 0.82–1.00). For the *ad libitum* meals, we excluded 12 out of 96 meals (revised average *R^2^* = 0.96; revised range = 0.81–1.00).

#### Satiation and Liking

Hunger, fullness, and liking ratings were elicited using a computer. In all other respects they were identical to those in Study 1. Appetite ratings were collected upon arrival at the laboratory (baseline), after participants had eaten the 400 kcal fixed portion, immediately after the *ad libitum* meal, and one hour later (immediately before the *ad libitum* snack meal).

#### Assessment of *Ad Libitum* Food Intake

Since fish, chips, and peas and beef stew with dumplings were both multi-component meals, we were concerned that participants might choose to eat different proportions of each component (e.g., fish, chips or peas). In response, we recorded both the total amount eaten and the amount eaten of each component, separately. For each component, we derived an estimate of energy intake (kcal) by multiplying the amount eaten (in g) by its energy density (kcal/g). We estimated the energy density of each component based on similar product packaging or published values [44]. Respectively, the energy density (kcal/g) of fish, chips and peas was estimated at 2.58, 1.85, and 0.68. The energy density (kcal/g) of beef stew and dumplings was estimated at 1.09 and 3.84, respectively.

#### 4.1.5. Procedure

Participants attended four test sessions on consecutive days. Each session was scheduled to start at 12:00 and lasted for 150 min. Participants were instructed to consume their normal breakfast and to abstain from eating and from consuming calorie-containing beverages for at least three hours before arrival at the laboratory. To assess compliance, participants reported the time since they last ate or consumed a calorie-containing beverage and completed baseline ratings of hunger and fullness. The experimenter then provided the participant with their fixed-portion test meal. Participants completed measures of liking and sensory characteristics. Video and covert weight recordings were then initiated simultaneously. Participants were instructed to eat the entire meal at their natural pace. Participants were also instructed to neither lean on the table while they ate nor move the plate or place cutlery on the plate or placemat. Immediately after eating, participants completed ratings of hunger and fullness. They were then presented with their *ad libitum* meal and were given the following instruction: “You will now have 20 min to eat as much or as little as you would like. You do not have to eat everything, eat until you feel comfortably full”. Output from the balance was recorded while participants consumed the *ad libitum* meal. After 20 min the participants rated their hunger and fullness for the third time. Participants then remained in the lab for a one-hour rest period, during which they watched non-emotive documentaries. They then completed another rating of hunger and fullness, and were offered access to the *ad libitum* snack meal for 20 min. At the end of the final session, participants completed the DEBQ and provided a written response to the question, “In your opinion, what was the purpose of this study”? Weight, height, and age were then recorded. Debriefing took place by email, after all of the data had been collected.

#### 4.1.6. Data Analysis

All data were analysed using IBM SPSS Statistics 21 (Armonk, NY, USA). Critical *p*-values of <0.05 were considered significant. One participant’s mouth was obscured in both video recordings of the fixed-portion slow meal. Therefore, these oral processing data were entered as missing. With a second participant we failed to record the weight of the separate components eaten in the *ad libitum* fish, chips and peas meal. These data were also entered as missing.

In the first instance, we assessed whether there were any baseline differences in the time since last eating, baseline fullness, or food temperature, across conditions. A 2 (fixed-portion meal type; fast and slow) × 2 (*ad libitum* meal type; same food and dessert) repeated-measures ANOVA revealed no significant difference in time since last eating and baseline fullness (see Table 5, all *p* > 0.33). There was no significant difference between the average temperature of fixed-portions of the fast and slow meals. However, fixed portions in the dessert condition were 4 °C hotter than in the same condition. Albeit statistically significant, the absolute magnitude of this difference is trivial. In addition, a paired-samples *t-*test revealed that there was no significant difference in the temperature of the *ad libitum* same meals (*t*(18) = 0.79, *p* = 0.44).

Second, we were interested to explore measures of oral processing during the fixed-portion meal. Each type of fixed-portion meal was consumed twice, once followed by the same meal and a second time followed by a dessert. Since the type of *ad libitum* meal was unknown to the participants while eating the fixed-portion meal we did not expect to see main or moderating effects of *ad libitum* meal type (same or dessert). Indeed, inspection of ICCs (two-way mixed-effects absolute agreement average measures) comparing responses across the same and dessert conditions indicated a high degree of correspondence (average ICC = 0.77). On this basis, for each measure of oral processing and for the slow and fast meal separately, we calculated a composite mean score by combining values across the same and dessert conditions. We then used a set of paired-samples *t*-tests to explore differences between the fast and slow meals. The same approach was used to analyse the change in fullness after these meals (average ICC = 0.61), together with their sensory characteristics (average ICC = 0.77) and associated ratings of liking (average ICC = 0.82). See Table 6 for mean values.

To explore differences in; (i) intake of the *ad libitum* meal (kcal), (ii) initial eating rate and change in eating rate of the *ad libitum* meals, (iii) fullness after eating the *ad libitum* meals, (iv) fullness one hour after eating the *ad libitum* meals, and (v) *ad libitum* intake of the snack meal, we again used 2 (fixed-portion meal type; fast and slow) × 2 (*ad libitum* meal type; same food and dessert) repeated-measures ANOVAs. Where we observed significant interactions between fixed-portion meal type and *ad libitum* meal type, planned pairwise comparisons were conducted between the fast and slow meal in each *ad libitum* meal type (same food or dessert), separately (Bonferroni corrected alpha level = 0.025).

Finally, Pearson’s correlation coefficients were calculated to identify specific oral processing characteristics that are reliable predictors of *ad libitum* intake. Specifically, we focused on relationships with; (i) oral processing measures in the fixed-portion meal and (ii) the initial eating rate and change in eating rate during the *ad libitum* meal.

### 4.2. Results

#### 4.2.1. Oral Processing Characteristics and Subjective Ratings

Table 6 summarises each oral processing measure for the fixed portions of fish, chips and peas (the slow meal) and for beef stew with dumplings (the fast meal). The slow food was eaten at a significantly slower rate (kcal/s), with more orosensory exposure (s), and a longer inter-bite interval (s). The slow food was also eaten with more chews per mouthful and with a smaller bite size (g). In all cases, these effects are highly significant. Across conditions, the slow meal was eaten at a slower initial rate (g/s). However, there was no statistically reliable difference in change in eating rate (g/s^2^). Table 5 shows mean values for measures of oral processing during the *ad libitum* meals.

Our fixed-portion test meals were rated as equally sweet, salty, savoury, firm, and chewy (see Table 6; all *p* > 0.5), suggesting that participants did not discriminate between the textural qualities of the two meals. Indeed, the only difference between our two fixed-portion meals was that beef stew with dumplings was rated as having a more intense flavour. The test foods were also rated as equally liked.

#### 4.2.2. Fullness after a Fixed Portion

Relative to baseline, participants reported a significantly greater increase in fullness (*t*(23) = 2.40, *p* = 0.03) after eating the slow meal than after eating the fast meal (see Table 6).

#### 4.2.3. *Ad Libitum* Intake

**Figure 3:**
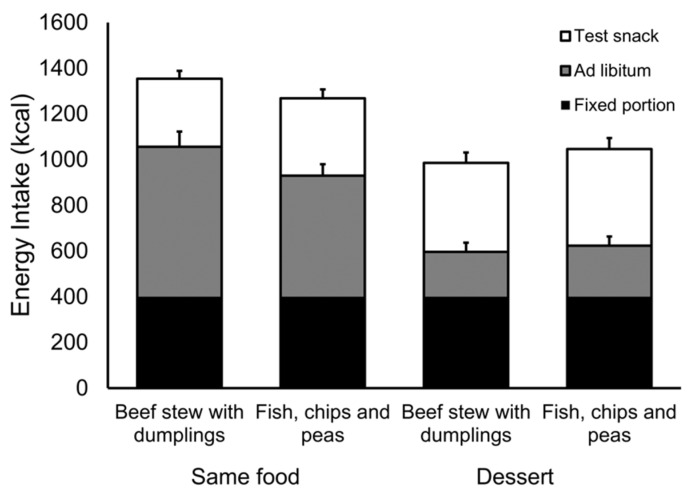
Mean (+S.E.) energy intake (kcal) of the fixed portion, *ad libitum* meal and snack food across conditions.

We found a significant main effect of fixed-portion meal type (fast and slow) on *ad libitum* food intake (kcal) (*F*(1, 22) = 5.35, *p* = 0.03, η_p_^2^ = 0.20). Participants consumed less food after eating a fixed portion of the slow meal (compared to eating a fixed portion of the fast meal). Energy intake was significantly greater in the same-food conditions than in the dessert conditions (*F*(1, 22) = 61.44, *p* < 0.001, η_p_^2^ = 0.74) and we found a significant interaction between fixed-portion meal type and *ad libitum* meal type (same and dessert) (*F*(1, 22) = 7.65, *p* = 0.01, η_p_^2^ = 0.26). Planned pairwise comparisons revealed that intake (kcal) of the *ad libitum* slow meal was 21.3% (126.3 kcal) lower than intake of *ad libitum* fast meal (*t*(22) = −3.18, *p* = 0.004). By contrast intake of the dessert was similar after consuming both fixed portion meals (*t*(23) = 0.95, *p* = 0.35). For associated means and standard errors see Figure 3.

#### 4.2.4. Relationships between Oral Processing Characteristics and *Ad Libitum* Intake

Table 7 summarises the relationships between oral processing of the fixed-portion meal and intake (kcal) at the *ad libitum* meal. Separate values are provided for the same food and the dessert conditions. In the dessert conditions, there were no significant correlations between oral processing of the fixed portion and intake of the dessert (all *p* > 0.09). Conversely, in the same-food conditions, several significant relationships were observed. For both fast and slow meals, greater *ad libitum* intake was associated with a faster initial eating rate (g/s), a faster overall rate (kcal/s), and with less orosensory exposure (s). Other variables also predicted *ad libitum* intake. However, these were not reliable across both types of fixed portion (see Table 7).

Table 7 also shows the relationships between *ad libitum* intake (kcal) and measures of eating rate during the *ad libitum* meals. A faster initial rate of eating (g/s) was associated with greater intake of the slow meals. Deceleration rate (g/s^2^) was not a significant predictor of *ad libitum* intake.

#### 4.2.5. Fullness after the *Ad Libitum* Meal

We explored differences in fullness immediately and one hour after the end of the *ad libitum* meal. Irrespective of fixed-portion meal type (fast or slow), similar fullness was reported, both immediately after eating (*F*(1, 23) = 0.61, *p* = 0.45, η_p_^2^ = 0.03) and one hour later (*F*(1, 23) = 0.34, *p* = 0.57, η_p_^2^ = 0.01). However, participants experienced greater fullness after consuming the *ad libitum* same meal than after consuming the *ad libitum* dessert. This was the case both at the end of their *ad libitum* meal (*F*(1, 23) = 17.39, *p* < 0.001, η_p_^2^ = 0.43) and one hour later (*F*(1, 23) = 29.83, *p* < 0.001, η_p_^2^ = 0.57). See Table 5 for the means and associated standard deviations.

#### 4.2.6. Snack Meal Intake

Figure 3 shows the mean (+S.E.) energy intake (kcal) of the snack meal one hour after the *ad libitum* meal. Intake was significantly greater after consuming the dessert (*F*(1, 23) = 11.64, *p* = 0.002, η_p_^2^ = 0.34). However, we found no significant difference based on having previously consumed the fast or slow meal (*F*(1, 23) = 1.80, *p* = 0.19, η_p_^2^ = 0.07). We conclude that there was relatively little compensation for the reduced intake of the slow food at this subsequent snack meal.

#### 4.2.7. Demand Awareness

Participants offered one or more explanations for the objectives of the study. Approximately half (46%) believed it was aimed at assessing the validity of appetite ratings as predictors of food intake. The same number (46%) mentioned effects of the taste of a food on subsequent food intake. A smaller proportion (30%) alluded to the effect of portion size on food intake and one participant mentioned mouth movements and their relation to the enjoyment of eating. Other participants proffered alternative suggestions but failed to reference the role of oral processing.

## 5. General Discussion

Study 1 demonstrated that across a range of unmodified hot meals, variation in oral processing is associated with differences in expected satiation and with fullness and satiety after the meals have been consumed. In Study 2 we compared two meals, one that is consumed faster than the other. They were otherwise matched for their liking, energy density, and portion size. Nevertheless, the slow meal produced greater fullness and it reduced subsequent food intake relative to the fast meal. We found little evidence that participants compensated for this difference in intake at a subsequent snack meal.

Our selection of foods in Study 2 was based on the assumption that they would promote different oral processing characteristics. From the outset we had a concern about “regression to the mean”. Specifically, that differences in oral processing observed in our sample in Study 1 might otherwise be attributed to chance and that these differences would not be replicated in Study 2. It was gratifying to see very similar effects preserved across studies and across pairs of *ad libitum* meal types (same or dessert). On this basis we are reasonably confident that differences in the oral processing of our test meals are reliable.

In Study 2 the effect of oral processing was only evident in the intake of the *ad libitum* meal when it was the same food as the fixed portion. Intake of the dessert was relatively small and we suspect that a dislike for this food masked the same effects of oral processing on intake. Consistent with this proposition, irrespective of fixed-portion meal type (fast or slow), participants reported relatively less fullness after consuming the dessert and consumed more at the *ad libitum* snack meal, one hour later. Alternatively, it may be relevant that participants were exposed to the slow and fast foods to a greater extent in the same conditions than in the dessert conditions. Thus, there was more opportunity for the differential effects of oral processing to be expressed. This cumulative effect (fixed portion + *ad libitum* meal) might explain why we saw significant differences in the same conditions but not in the dessert conditions.

Estimates suggest that a reduction of 100 kcal per day would be sufficient to prevent weight gain in 90% of the population [77]. Although we note that the exact value of this kilocalorie reduction per day will vary considerably between individuals [78]. Our observed effect of oral processing on intake was modest (6.5% across the entire 150 min test session—86 kcal). Nevertheless, it would be sufficient to achieve this target. Broadly, this approach is consistent with the idea of a dietary nudge—the prospect that a small reduction in energy intake (between 8% and 16% per day) can be implemented by stealth and can have a considerable impact on health [15,16]. In this respect, we note that the effects were observed when both male and female participants were included in the sample and that both of the foods in Study 2 were equally liked. Moreover, they were rated as having the same textural qualities (hardness, chewiness *etc.*), which suggests that awareness of these differences is not a prerequisite for a change in behaviour. Recent research has demonstrated that it might be possible to introduce subtle modifications to recipes or formulations to promote particular aspects of oral processing (e.g., orosensory exposure) [24,25]. Again, this might be achieved covertly and it contrasts a strategy based on reductions in energy density. At a population level, this might represent a viable target for food manufacturers to help to nudge their consumers to reduce energy intake. Further research is needed to establish that modifications to foods that promote particular aspects of oral processing have a reliable effect on behaviour. This work might consider the role of meal variety, flavour intensity, flavour complexity, and unit size. In addition, future research should consider the extent to which people adapt to manipulations in oral processing. In particular, our studies raise questions about compensation over longer periods, over a day or more. Based on previous observations [17] we suspect this is unlikely. However, this remains to be determined.

Our primary concern was to investigate whether, and irrespective of individual differences in oral processing, specific oral processing variables are reliably associated with expected satiation, post-meal fullness, and satiety, across a range of foods. Nevertheless, we recognise that our measures of oral processing could equally be applied to the study of individual differences. Specifically, an alternative approach might be to assess a larger number of participants on a limited number of foods and to explore which oral processing behaviours are the best predictors of expected satiation, post-meal fullness, and satiety, within each food separately. It would also be interesting to explore whether particular individuals are more responsive to the effects of oral processing. This type of analysis was beyond the scope of our study but represents an interesting avenue for future research.

In this work we are not making strong claims about a causal role for oral processing in satiation and satiety. Our observations are primarily correlational in nature. Nevertheless, in this context, and as we note earlier, there is very strong independent evidence implicating oral processing as a causal determinant of satiation and satiety [21,79,80]. For recent reviews see Robinson *et al.* [33] and Hogenkamp and Sciöth [34].

Finally, this work raises questions about the underlying mechanism that supports these effects of oral processing. Historically, researchers have tended to refer to a general concept of “oral metering”—the mouth monitoring the arrival of nutrients [81]. However, the precise cognitive and physiological event(s) that trigger this metering remain unclear and this merits continued investigation. More generally, de Graaf [22] has argued that the taste system updates the brain about the inflow of nutrients via cephalic phase responses (CPRs). CPRs are physiological responses that prepare the body for the arrival of nutrients. In future, it would be interesting to look at naturally occurring relationships between CPRs and specific oral processing behaviours. In particular, this index of CPRs might take the form of a thermic response to the food [82]. An alternative or additional possibility is that oral processing affects memory for recent eating [83,84] by promoting better encoding of a food that is eaten at a slower rate [85]. These ideas are currently being explored in ongoing work in our group.

## 6. Conclusions

Together, our findings suggest that variations in oral processing across a range of unmodified everyday meals can affect fullness after consuming a fixed portion (Study 1) and can also impact meal size (Study 2). Modifying food form to encourage increased oral processing (albeit to a lesser extent than in experimental manipulations) might represent a viable target for food manufacturers to help to nudge consumers to manage their weight.

## Figures and Tables

**Table 1:** Portion size and macronutrient composition for the 400-kcal portions of the 20 test meals ^a^. Respectively, *^L^ and *^H^ indicates that the test meal belongs to the low- and high- energy-dense subgroups.

	Test Meal	Portion Size (g)	Energy Density (kcal/g)	Protein (g)	Carbohydrate (g)	Fat (g)	Fibre (g)
*^H^	Beef cannelloni	227.0	1.8	17.2	38.4	18.4	5.6
*^H^	Beef lasagne	219.9	1.8	18.1	34.7	21.0	4.3
*^H^	Beef stew with dumplings	219.7	1.8	19.5	33.9	17.4	14.5
*^H^	Chicken and bacon pasta bake	221.7	1.8	19.3	33.2	20.6	2.5
	Chicken and prawn paella—version 1	297.3	1.4	24.4	54.4	8.9	4.5
	Chicken and prawn paella—version 2	332.3	1.2	27.8	47.7	9.2	5.4
	Chicken chow mein	322.3	1.2	21.0	40.6	15.2	8.4
*^L^	Chicken in barbeque sauce with potato wedges	301.7	1.3	29.0	45.3	10.2	5.4
*^L^	Fish pie	320.4	1.3	26.7	37.3	15.0	4.1
*^H^	Fish, chips and peas	222.9	1.8	12.5	50.0	15.0	6.9
	Lancashire hotpot	321.4	1.2	26.4	36.6	14.4	10.2
	Macaroni cheese	204.1	2.0	16.9	41.5	17.5	4.1
*^L^	Mushroom risotto	299.7	1.3	7.1	51.8	17.8	1.8
	Pepperoni pizza	127.9	3.1	18.9	37.2	18.8	3.1
*^L^	Roast chicken dinner	308.0	1.3	32.8	46.5	7.8	6.2
	Sausage and mashed potato	285.2	1.4	15.4	38.9	18.7	7.5
	Spaghetti carbonara	236.5	1.7	13.4	43.5	18.4	3.3
	Sweet and sour chicken with rice	251.2	1.6	19.2	58.8	8.0	6.2
	Thai green chicken curry with rice	259.1	1.5	19.4	38.1	17.6	6.1
	Three cheese omelette	169.6	2.4	20.7	10.3	30.5	1.5
*^L^	Vegetable biryani	315.9	1.3	9.1	50.7	15.1	12.3

^a^ Each test meal was cooked according to the manufacturer’s recommendations and the total cooked weight (without packaging) was recorded. The energy density (kcal/g) and macronutrient composition of each test meal was calculated by dividing the number of calories, amount of protein, amount of carbohydrate, amount of fat, and amount of fibre reported on the product packaging (per pack cooked as per instructions) by the recorded cooked weight. All values are rounded to one decimal place.

**Table 2:** Mean (±S.D.) scores of time since last eating (mins), baseline fullness (mm), food temperature (°C), expected satiation (kcal), liking (mm), fullness after eating a fixed portion (mm), satiety (fullness area under the curve (AUC)) and oral processing behaviours across the 20 test meals.

Test Meal	Time Since Last Eating (Mins)	Baseline Fullness (mm)	Food Temperature (°C)	Expected Satiation (kcal)	Liking (mm)	Fullness (mm)	Satiety (AUC)	Eating Rate (kcal/s)	Initial Eating Rate (g/s)	Change in Eating Rate (g/s^2^)	Orosensory Exposure Time (s)	Inter-Bite Interval (s)	Average Bite Size (g)	Bite Rate (per s)	Number of Chews (per Mouthful)
Beef cannelloni	363.6 (316.4)	21.4 (18.3)	86.2 (10.0)	350.9 (107.2)	76.4 (21.3)	78.5 (16.2)	182.7 (65.1)	1.6 (0.5)	0.8 (0.3)	−0.00003 (0.00050)	202.7 (106.2)	75.6 (35.8)	9.6 (2.6)	0.095 (0.030)	7.8 (4.4)
Beef lasagne	369.7 (271.4)	25.9 (18.6)	82.9 (4.1)	382.2 (97.9)	75.2 (15.3)	72.5 (20.4)	183.3 (74.7)	1.7 (0.5)	0.9 (0.3)	−0.00012 (0.00063)	197.4 (133.9)	73.3 (19.3)	9.3 (1.4)	0.101 (0.029)	7.6 (5.3)
Beef stew with dumplings	339.5 (210.6)	22.2 (19.3)	78.2 (7.8)	369.5 (111.2)	67.9 (18.6)	68.0 (18.8)	156.5 (66.7)	2.0 (0.6)	1.1 (0.6)	−0.00084 (0.00177)	169.9 (78.9)	48.9 (25.7)	11.3 (1.6)	0.099 (0.035)	8.6 (4.1)
Chicken and bacon pasta bake	430.0 (294.4)	20.3 (18.4)	79.3 (8.4)	357.9 (124.9)	77.1 (15.7)	75.1 (21.8)	190.3 (60.0)	1.6 (0.5)	0.8 (0.2)	−0.00001 (0.00044)	217.2 (107.3)	55.1 (23.4)	9.0 (2.5)	0.105 (0.040)	9.5 (5.3)
Chicken and prawn paella	395.9 (313.7)	17.6 (13.2)	77.6 (7.2)	543.6 (114.0)	75.4 (20.7)	88.5 (7.5)	196.2 (67.1)	1.1 (0.4)	0.9 (0.3)	−0.00009 (0.00037)	327.1 (144.5)	78.5 (33.4)	9.4 (2.6)	0.097 (0.031)	10.5 (3.6)
Chicken chow mein	268.2 (157.8)	20.7 (14.4)	78.9 (8.6)	532.2 (135.1)	75.1 (20.4)	85.4 (11.8)	210.8 (45.9)	1.0 (0.3)	0.8 (0.3)	−0.00018 (0.00055)	324.1 (122.5)	90.1 (35.9)	10.9 (3.0)	0.080 (0.022)	11.4 (5.1)
Chicken in barbecue sauce with wedges	370.0 (276.2)	30.1 (15.2)	74.4 (5.9)	445.0 (128.3)	67.7 (17.0)	80.6 (20.7)	203.4 (65.5)	1.2 (0.3)	0.8 (0.2)	−0.00030 (0.00043)	283.1 (78.4)	80.9 (23.3)	9.9 (2.0)	0.088 (0.016)	9.6 (2.8)
Fish pie	428.6 (275.6)	18.4 (16.7)	82.9 (8.4)	427.0 (55.4)	65.7 (20.2)	83.1 (11.1)	195.0 (55.2)	1.2 (0.4)	0.9 (0.3)	−0.00004 (0.00058)	278.5 (128.2)	92.6 (33.1)	10.5 (2.3)	0.090 (0.022)	8.6 (3.3)
Fish, chips and peas	267.3 (152.3)	14.5 (11.1)	80.6 (11.2)	411.0 (117.5)	71.8 (17.2)	76.6 (17.5)	190.2 (75.4)	1.0 (0.2)	0.6 (0.1)	−0.00020 (0.00024)	357.7 (126.4)	67.3 (33.2)	5.9 (1.5)	0.098 (0.026)	10.7 (3.1)
Lancashire hotpot	311.8 (237.2)	19.4 (16.7)	76.9 (8.4)	467.6 (94.6)	73.0 (22.8)	88.1 (10.1)	215.7 (47.3)	1.3 (0.3)	1.0 (0.3)	−0.00020 (0.00057)	238.3 (106.6)	86.2 (38.4)	11.1 (2.7)	0.097 (0.024)	8.4 (3.5)
Macaroni cheese	425.5 (301.7)	23.5 (21.3)	79.6 (5.5)	282.1 (119.1)	59.5 (16.4)	63.7 (23.0)	173.8 (74.7)	1.7 (0.7)	0.9 (0.5)	−0.00064 (0.00109)	224.5 (136.6)	55.1 (28.2)	7.8 (1.7)	0.113 (0.044)	8.8 (5.3)
Mushroom risotto	311.4 (166.8)	14.6 (11.1)	80.4 (7.8)	391.0 (89.8)	68.0 (23.5)	82.8 (13.1)	196.3 (50.5)	1.3 (0.5)	0.9 (0.5)	0.00012 (0.00115)	264.5 (147.7)	84.3 (31.1)	9.9 (1.8)	0.102 (0.036)	9.0 (5.2)
Pepperoni pizza	332.0 (241.6)	17.7 (11.0)	76.5 (12.7)	284.7 (152.6)	78.2 (16.1)	65.0 (24.0)	171.5 (69.5)	1.5 (0.6)	0.4 (0.2)	0.00052 (0.00155)	255.8 (128.5)	48.9 (18.9)	7.4 (1.7)	0.065 (0.015)	16.6 (5.4)
Roast chicken dinner	305.0 (191.9)	21.6 (14.6)	77.7 (9.7)	545.0 (163.3)	72.6 (16.0)	86.6 (9.0)	194.4 (68.8)	1.0 (0.3)	0.7 (0.2)	−0.00009 (0.00027)	328.1 (133.3)	94.0 (38.2)	8.7 (2.9)	0.095 (0.024)	9.2 (2.2)
Sausage and mashed potato	339.5 (266.0)	22.1 (16.6)	81.3 (7.9)	396.5 (95.8)	78.7 (16.5)	79.0 (13.5)	205.6 (53.8)	1.7 (0.4)	1.3 (0.4)	−0.00091 (0.00087)	169.0 (74.0)	83.7 (25.3)	13.0 (2.8)	0.093 (0.019)	6.3 (2.5)
Spaghetti carbonara	444.1 (333.9)	14.9 (15.5)	75.7 (7.3)	362.1 (96.4)	73.0 (13.2)	79.7 (12.6)	188.2 (57.7)	1.5 (0.4)	0.8 (0.2)	0.00030 (0.00067)	225.4 (116.0)	65.7 (21.0)	10.7 (3.5)	0.087 (0.026)	9.6 (6.1)
Sweet and sour chicken with rice	436.6 (325.1)	17.1 (11.8)	78.4 (11.2)	419.7 (103.8)	68.5 (24.7)	76.7 (13.6)	185.7 (58.9)	1.5 (0.4)	0.9 (0.4)	−0.00045 (0.00118)	251.1 (120.8)	50.0 (26.7)	10.4 (2.6)	0.092 (0.029)	10.5 (4.1)
Thai green curry with rice	362.3 (281.6)	27.5 (18.6)	76.5 (7.3)	389.0 (101.1)	74.9 (14.1)	81.0 (12.7)	200.3 (50.8)	1.4 (0.4)	0.9 (0.4)	−0.00041 (0.00135)	254.2 (125.2)	66.3 (24.7)	10.1 (2.4)	0.092 (0.034)	10.1 (4.9)
Three cheese omelette	330.9 (214.2)	24.7 (22.7)	75.9 (4.9)	209.6 (93.7)	41.8 (16.6)	73.1 (17.4)	159.9 (62.9)	1.7 (0.7)	0.7 (0.3)	−0.00019 (0.00041)	233.4 (120.8)	63.6 (33.0)	8.5 (2.4)	0.084 (0.034)	12.9 (6.3)
Vegetable biryani	292.1 (205.4)	14.6 (9.6)	78.1 (9.1)	570.3 (144.6)	71.6 (14.1)	86.5 (13.4)	208.3 (55.7)	0.9 (0.3)	0.9 (0.3)	−0.00052 (0.00035)	426.9 (189.8)	72.7 (33.2)	7.8 (2.0)	0.093 (0.026)	12.2 (4.7)
*F*	0.7	1.7	1.2	10.0	3.7	3.2	2.0	13.5	6.5	1.8	14.3	4.3	10.5	4.8	7.9
*df*_within_	19	19	19	19	19	19	19	19	19	19	19	19	19	19	19
*df*_error_^a^	190	190	171	190	190	190	190	190	190	190	190	190	190	190	190
*P*	0.821	0.041	0.267	0.000	0.000	0.000	0.010	0.000	0.000	0.023	0.000	0.000	0.000	0.000	0.000

^a^ *df*_error_ values vary across measures since SPSS excludes anyone with a missing value list wise.

**Table 3:** Inter-relationships (Pearson’s partial correlations) between the oral processing variables ^a^.

	Eating Rate (kcal/s)	Initial Eating Rate (g/s)	Change in Eating Rate (g/s^2^)	Orosensory Exposure Time (s)	Inter-Bite Interval (s)	Bite Rate (per s)	Number of Chews (Per Mouthful)	Average Bite Size (g)
Eating rate (kcal/s)	-							
Initial eating rate (g/s)	0.24	-						
Change in eating rate (g/s^2^)	−0.14	−0.74 ***	-					
Orosensory exposure time (s)	−0.92 ***	−0.33	0.03	-				
Inter-bite interval (s)	−0.65 **	0.20	0.07	0.39	-			
Bite rate (per s)	0.16	0.42	−0.34	−0.15	−0.02	-		
Number of chews (per mouthful)	−0.17	−0.73 ***	0.38	0.38	−0.39	−0.68 **	-	
Average bite size (g)	0.26	0.77 ***	−0.31	−0.47 *	0.27	−0.08	−0.52 *	-

**^a^** Baseline fullness was included as a controlling factor in these analyses. * *p* < 0.05; ** *p* < 0.01; *** *p* < 0.001. All values are rounded to two decimal places.

**Table 4:** In each subgroup, relationships (Pearson’s partial correlations) between oral processing characteristics and expected satiation (kcal), post-meal fullness (mm) and satiety (AUC) ^a^.

	Low Energy Dense kcal/g = 1.3			High Energy Dense kcal/g = 1.8		
	Expected Satiation (kcal)	Fullness (mm)	Satiety (AUC)	Expected Satiation (kcal)	Fullness (mm)	Satiety (AUC)
Eating rate (kcal/s)	−0.99 **	−0.56	−0.90	−0.38	−0.48	−0.80
Initial eating rate (g/s)	−0.58	−0.90	−0.14	−0.31	−0.53	−0.86
Change in eating rate (g/s^2^)	−0.79	0.01	−0.98 *	−0.08	0.79	0.99 **
Orosensory exposure time (s)	0.97 *	0.38	0.96 *	0.71	0.08	0.56
Inter-bite interval (s)	−0.49	0.39	−0.84	0.20	0.57	0.58
Bite rate (Per s)	−0.18	0.60	−0.62	−0.46	−0.01	0.09
Number of chews (Per mouthful)	0.74	−0.08	0.94	0.46	−0.41	0.16
Average bite size (g)	−0.93	−0.55	−0.79	−0.52	−0.30	−0.76

^a^ Baseline fullness was included as a controlling factor in these analyses. * *p* < 0.05; ** *p* < 0.01; *** *p* < 0.001. All values are rounded to two decimal places.

**Table 5:** Mean (*±*S.D.) scores for baseline measures, *ad libitum* meal oral processing and fullness after the *ad libitum* meal.

	Same Condition		Dessert Condition		ANOVA		
	Beef Stew with Dumplings	Fish, Chips and Peas	Beef Stew with Dumplings	Fish, Chips and Peas	Main Effect of Fixed-Portion Meal Type (*F* Ratio)	Main Effect of *ad Libitum* Meal Type (*F* Ratio)	Interaction (*F* Ratio)
*Baseline measures*							
Time since last eating (mins)	374.6 (298.4)	389.6 (277.3)	403.3 (286.0)	396.3 (273.6)	0.01	0.28	0.14
Baseline fullness (mm)	27.0 (18.1)	26.0 (15.6)	28.5 (25.0)	23.7 (14.4)	1.01	0.03	0.30
Fixed portion food temperature (°C)	71.8 (5.8)	73.0 (5.2)	76.4 (8.3)	76.5 (7.6)	0.23	7.36 *	0.16
							
*Ad libitum meal oral processing*							
Initial eating rate (g/s)	0.74 (0.22)	0.59 (0.17)	0.46 (0.22)	0.59 (0.43)	0.02	2.47	3.01
Change in eating rate (g/s^2^)	−0.0004 (0.0003)	−0.0002 (0.0002)	−0.0014 (0.0034)	−0.0007 (0.0012)	0.50	1.81	0.21
							
*Fullness ratings*							
Immediately after eating the *ad libitum* meal (mm)	88.9 (10.9)	89.1 (6.4)	71.9 (21.0)	75.3 (16.6)	0.61	17.39 ***	0.70
One hour after eating the *ad libitum* meal (mm)	72.7 (18.0)	74.6 (10.7)	56.5 (21.9)	57.8 (15.1)	0.34	29.83 ***	0.01

* *p* < 0.05; ** *p* < 0.01; *** *p* < 0.001.

**Table 6:** Mean (±S.D.) scores for oral processing characteristics, sensory ratings, change in fullness, and liking across the fixed portion meals.

	Beef Stew with Dumplings	Fish, Chips and Peas	*t*	*df*	*p*
*Oral processing behaviour*					
Eating rate (kcal/s)	1.95 (0.75)	1.16 (0.46)	−8.2	22	<0.001
Orosensory exposure time (s)	166.0 (61.1)	310.4 (140.3)	7.0	22	<0.001
Inter-bite interval (s)	76.2 (45.3)	100.5 (59.3)	2.7	22	0.01
Bite rate (Per s)	0.10 (0.03)	0.10 (0.03)	0.9	22	0.36
Number of chews (Per mouthful)	8.3 (3.3)	9.9 (2.8)	3.1	22	0.005
Average bite size (g)	11.4 (3.2)	6.5 (1.8)	−9.8	22	<0.001
Change in eating rate (g/s^2^)	−0.0001 (0.0020)	−0.0003 (0.0004)	−0.5	18	0.66
Initial eating rate (g/s)	0.93 (0.47)	0.66 (0.32)	−2.9	18	0.01
*Sensory ratings*					
Flavour intensity (mm)	58.0 (19.7)	48.1 (13.6)	−2.2	23	0.04
Sweetness (mm)	23.9 (14.7)	22.8 (16.1)	−0.5	23	0.65
Saltiness (mm)	53.1 (17.0)	51.2 (21.9)	−0.6	23	0.57
Savoury (mm)	70.0 (15.2)	68.2 (15.4)	−0.6	23	0.56
Firmness (mm)	40.7 (21.1)	41.1 (19.4)	0.1	23	0.89
Chewiness (mm)	56.2 (23.4)	54.9 (24.8)	−0.6	23	0.53
Change in fullness (mm)	31.8 (17.9)	38.4 (17.0)	2.4	23	0.03
Liking (mm)	66.3 (19.5)	59.1 (19.4)	−1.4	23	0.18

**Table 7:** Relationships (Pearson’s) between *ad libitum* intake (kcal) and measures of oral processing in the fixed-portion and the *ad libitum* meals.

	Same Condition Intake (kcal)		Dessert Condition Intake (kcal)	
	Beef Stew with Dumplings	Fish, Chips and Peas	Beef Stew with Dumplings	Fish, Chips and Peas
*Oral processing in the fixed portion meal*				
Eating rate (kcal/s)	0.59 **	0.62 **	0.11	0.17
Orosensory exposure time (s)	−0.55 **	−0.63 **	−0.04	−0.26
Inter-bite interval (s)	−0.46 *	−0.34	0.05	−0.15
Bite rate (Per s)	0.24	0.44 *	0.06	0.11
Number of chews (Per mouthful)	−0.13	−0.58 **	−0.07	−0.25
Average bite size (g)	0.52 **	0.29	0.08	0.25
Initial eating rate (g/s)	0.67 **	0.61 **	0.35	−0.03
Deceleration rate (g/s^2^)	−0.27	−0.28	−0.28	0.01
*Oral processing in the ad libitum meal*				
Initial eating rate (g/s)	0.35	0.55 **	0.40	0.53 *
Deceleration rate (g/s^2^)	0.15	−0.38	0.00	−0.43

* *p* < 0.05; ** *p* < 0.01; *** *p* < 0.001.

## Supplementary Materials

The following are available online at http://www.mdpi.com/2072-6643/8/5/315/s1, Figure S1: Images of the 400-kcal portions for each of the 20 test meals, as served, Text S1: Script in Matlab to process the continuous meal-weight data, Table S1: Absolute values of the SMDs for oral processing. Values are provided for each food pair in the low and high energy-dense subgroups, separately. Respectively, *^L^ and *^H^ indicates that the test meal belongs to the low- and high- energy dense subgroup.
