# Supplementary material for: Variation in the Oral Processing of Everyday Meals Is Associated with Fullness and Meal Size; A Potential Nudge to Reduce Energy Intake?
Source: Nutrients. 2016 May 21;8(5):315. doi: 10.3390/nu8050315 (PMC4882727; doi:10.3390/nu8050315)
Supplement: Supplementary file 1 [file nutrients-08-00315-s001.pdf]

Supplementary Materials: Variation in the Oral Processing of Everyday Meals is Associated with Fullness and Meal Size; A Potential Nudge to Reduce Energy Intake?

Danielle Ferriday, Matthew L. Bosworth, Nicolas Godinot, Nathalie Martin, Ciarán G. Forde, Emmy Van Den Heuvel, Sarah L. Appleton, Felix J. Mercer Moss, Peter J. Rogers and Jeffrey M. Brunstrom

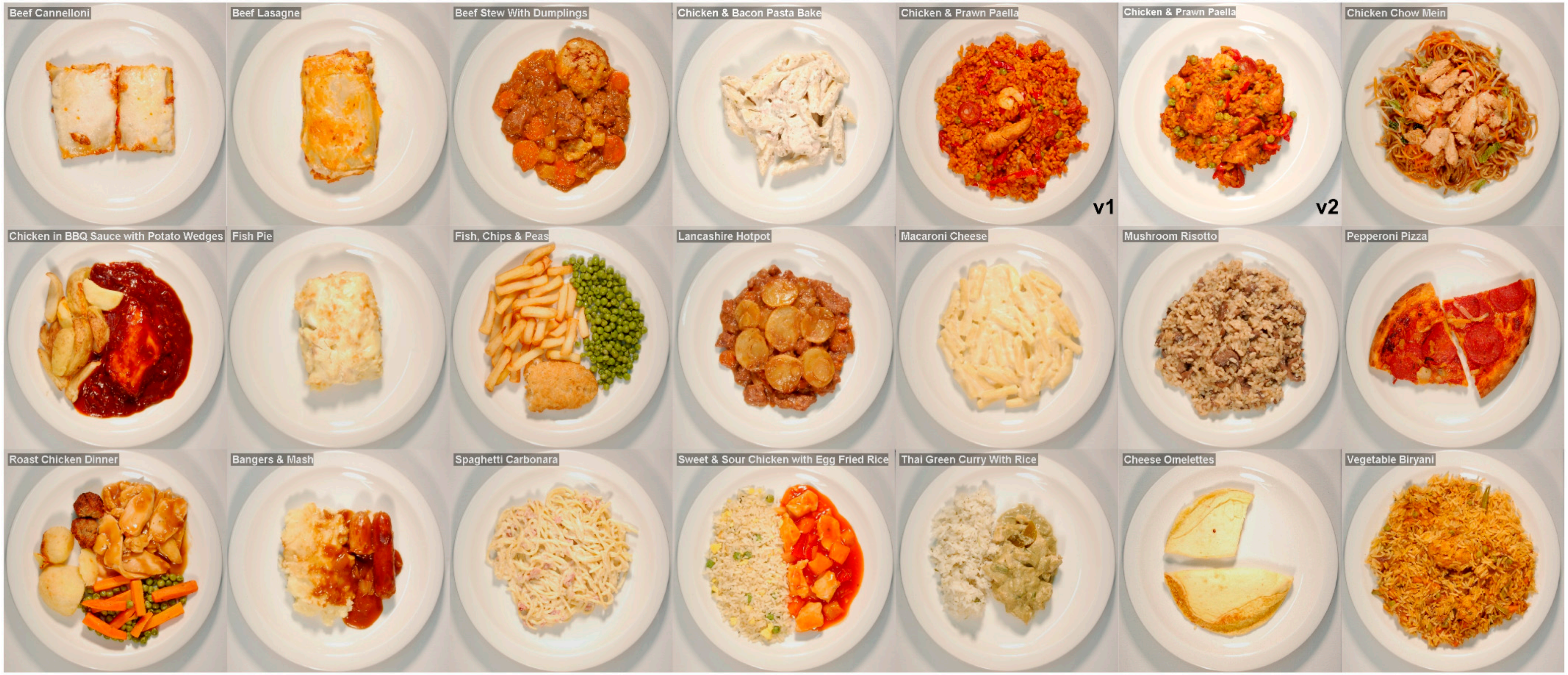

Figure S1. Images of the 400-kcal portions for each of the 20 test meals, as served.

Text S1. Script in Matlab to process the continuous meal-weight data.

```
%Matlab code example for Study 1 fixed portion meals - functions appear
at the end

%Experimenter defines the start and end point of the meal

load('Raw data file location')

for i = 1:12
      for j = 1:20
      d = mark_points1(raw_weight_data {i,j});
      startX(i,j) = d(1,1);
      endX(i,j) = d(1,2);
      save(['Save location'], 'startX', 'endX');
    end
end

%Then the raw data is trimmed, filtered, plotted, inverted to form a CIC
and a quadratic equation is fitted.

load('Raw data file location')
load('Marked data file with start and end points of the meal')

for i = 1:12
      for j = 1:20

        trimmed_data = trim_weight(raw_weight_data {i,j},
        round(startX(i,j)), round(endX(i,j)));
        cic = filter_weight2(trimmed_data)';
        cic = -cic;
        cic = cic-cic(1); %

        [fitresult, gof1] = createFit([1:length(cic)], cic,1);
        Coeff_1(i,j) = fitresult.p1;
        Coeff_22(i,j) = fitresult.p2;
        Coeff_3(i,j) = fitresult.p3;
```

```
     end
end

%createFit

function [fitresult, gof] = createFit(x, data2,disp)

%% Fit: 'untitled fit 1'.
[xData, yData] = prepareCurveData( x, data2 );

% Set up fittype and options.
ft = fittype( 'poly2' );
opts = fitoptions( ft );
opts.Lower = [-Inf -Inf -Inf];
opts.Upper = [Inf Inf Inf];

% Fit model to data.
[fitresult, gof] = fit( xData, yData, ft, opts );

%filter_weight2 function

function data = filter_weight2(data)



inc = 0;
dec = 0.025;

data = medfilt1(data,3);

for i = 1:length(data)-1
    if (data(i+1)-data(i))>inc
       data(i+1) = data(i);
    end
    if (data(i)-data(i+1))>dec
```

```
        data(i+1) = data(i);
    end
end


%mark_points function

function data = mark_points1(I)

toff=2;
P=zeros(2,9,1);
imgs=1;
i=1;
bb=[1 size(I,2) 1 size(I,1)];
o=1;
drw=1;
while 1
    if drw
        ph=zeros(9,1);
        th=zeros(9,1);

        plot(I);
        hold on;
        for j=1:size(P,3)
            if j~=o
                for k=1:9
                    if all(P(:,k,j))
                        plot(P(1,k,j),P(2,k,j),'g.','markersize',5);
                    end
                end
            end
        end
        for j=1:9
            if all(P(:,j,o))
                v='on';
```

```
            else
                v='off';
            end

ph(j)=line(P(1,j,o),P(2,j,o),'color','r','marker','x','visible',v);

th(j)=text(P(1,j,o)+toff,P(2,j,o)+toff,sprintf('%d',j),'color','r','visib
le',v);
        end
        hold off;
title(sprintf('%d/%d object %d',i,length(imgs),o));
        drw=0;
    end
    while 1
        [x,y,b,t]=vgg_event_wait;
        if t==2
            break
        end
    end
    if b>='1'&b<='9'
        p=b-'1'+1;
        if all([x;y]==P(:,p,o))
            P(:,p,o)=0;
            set(ph(p),'visible','off');
            set(th(p),'visible','off');
            drawnow;
        else
            P(:,p,o)=[x;y];
            set(ph(p),'xdata',P(1,p,o),'ydata',P(2,p,o),'visible','on');
            set(th(p),'position',[P(1,p,o)+toff P(2,p,o)+toff
0],'visible','on');
            drawnow;
        end
    elseif b=='b'
        P(:)=0;
        for p=1:9
            set(ph(p),'visible','off');
```

```
            set(th(p),'visible','off');
        end
    elseif b=='z'
        w=(bb(2)-bb(1)+1)/4;
        h=(bb(4)-bb(3)+1)/4;
        bb=[max(x-w,1) min(x+w,size(I,2)) max(y-h,1) min(y+h,size(I,1))];
        axis(bb);
    elseif b=='x'
        bb=[1 size(I,2) 1 size(I,1)];
        axis(bb);
    elseif b=='w'
        o=o+1;
        if o>size(P,3)
            P(:,:,o)=0;
        end
        drw=1;
        %              bb=[1 size(I,2) 1 size(I,1)];
    elseif b=='q'
        o=max(o-1,1);
        drw=1;
        %              bb=[1 size(I,2) 1 size(I,1)];
    elseif b=='n'||b==' '
        i=i+1;
        break
    elseif b=='p'||b=='p'
        i=i-1;
        break
    end
    data = P;
end



%trim_weight function

function data = trim_weight(data,st,en);
```

```
if en<length(data)
data(en+1:end) = [];
end

if st>1
data(1:st-1) = [];
end

%vgg_event_wait function

function [x,y,button,type] = vgg_event_wait(callback_arg, callback_arg2)

global VGG_EVENT_WAIT_E VGG_EVENT_WAIT_DOWN VGG_EVENT_WAIT_HANDLE

if isempty(VGG_EVENT_WAIT_DOWN)
  VGG_EVENT_WAIT_DOWN = 0;
end

timeout = Inf; % Timeout in seconds

if nargin ~= 1
  main_entry = 0;
  if nargin == 0
    main_entry = 1;
  end
  if nargin == 2
    % param/value
    main_entry = 1;
    switch callback_arg
     case 'timeout', timeout = callback_arg2;
     otherwise
      error(['bad parm/value pair']);
    end
  end
```

```
% The main entry point.
% 1. Set up handlers
% 2. Loop waiting for press
% 3. Collect event information and return
if main_entry
  interruptible = get(gcf, 'interruptible');
  busyaction = get(gcf, 'busyaction');
  % Make sure that the Motion function doesn't interrupt itself
  set(gcf, 'Interruptible', 'off', 'busyaction', 'queue');


  % User entry point
  motion_fcn = get(gcf, 'WindowButtonMotionFcn'); set(gcf,
'WindowButtonMotionFcn', 'vgg_event_wait(''motion'')');
  down_fcn = get(gcf, 'WindowButtonDownFcn'); set(gcf,
'WindowButtonDownFcn', 'vgg_event_wait(''down'')');
  up_fcn = get(gcf, 'WindowButtonUpFcn'); set(gcf, 'WindowButtonUpFcn',
'vgg_event_wait(''up'')');
  keypress_fcn = get(gcf, 'KeyPressFcn'); set(gcf, 'KeyPressFcn',
'vgg_event_wait(''key'')');


  VGG_EVENT_WAIT_HANDLE = line(nan,nan, 'visible', 'off', 'userdata',
0);
  VGG_EVENT_WAIT_E = [0 0 0 0];
  error_occurred = 0;
  lasterr('')
  if timeout == Inf
    while ~any(VGG_EVENT_WAIT_E)
  try
    waitfor(VGG_EVENT_WAIT_HANDLE, 'userdata');
  catch
    error_occurred = 1;
    break
  end
    end
  else
    pause(timeout);
    if ~any(VGG_EVENT_WAIT_E)
```

```
    VGG_EVENT_WAIT_E = [inf inf inf inf];
      end
    end

    x = VGG_EVENT_WAIT_E(1);
    y = VGG_EVENT_WAIT_E(2);
    button = VGG_EVENT_WAIT_E(3);
    type = VGG_EVENT_WAIT_E(4);

    % Clear motion function
    set(gcf, 'WindowButtonMotionFcn', '');
    set(gcf, 'WindowButtonDownFcn', '');
    set(gcf, 'WindowButtonUpFcn', '');
    set(gcf, 'KeyPressFcn', '');
    set(gcf, 'interruptible', interruptible);
    set(gcf, 'busyaction', busyaction);

    delete(VGG_EVENT_WAIT_HANDLE);
    clear global VGG_EVENT_WAIT_E VGG_EVENT_WAIT_HANDLE
    if error_occurred
      error(['vgg_event_wait: PASSTHRU: [' lasterr ']']);
    end

    if nargout <= 1
      x = [x y button type];
    end

    return
  end
end

if nargin == 1
  if isstr(callback_arg)
    switch callback_arg
     case 'clear'
```

```
      VGG_EVENT_WAIT_DOWN = 0;
      VGG_EVENT_WAIT_E = [0 0 0 0];
      set(gcf, 'WindowButtonMotionFcn', '');
      set(gcf, 'WindowButtonDownFcn', '');
      set(gcf, 'WindowButtonUpFcn', '');
      set(gcf, 'KeyPressFcn', '');

      % Empty the queue
      set(0, 'busyaction', 'cancel');
      set(gcf, 'busyaction', 'cancel');
      set(gca, 'busyaction', 'cancel');
      pause(0.01)
      return
     case 'motion'
     case 'key'
     case 'up'
     case 'down'

     otherwise
      error(['unknown usage: vgg_event_wait ' callback_arg]);
    end
  end
end

%%%%% Callback entry point
% Get XY pos
p = get(gca, 'currentpoint');
p = [p(1,1) p(1,2)];

% Find which mouse button is pressed
button = get(gcf, 'SelectionType');
if strcmp(button,'open')
  button = 4;
elseif strcmp(button,'normal')
  button = 1;
```

```
elseif strcmp(button,'extend')
  button = 2;
elseif strcmp(button,'alt')
  button = 3;
else
  error('Invalid mouse selection.')
end

VERBOSE = 0;

% fill waiting VGG_EVENT_WAIT_E struct
if strcmp(callback_arg, 'motion')
  if VERBOSE, fprintf('.'); end
  VGG_EVENT_WAIT_E = [p button*VGG_EVENT_WAIT_DOWN 0];
elseif strcmp(callback_arg, 'down')
  if VERBOSE, fprintf('d'); end
  VGG_EVENT_WAIT_E = [p button 1];
  VGG_EVENT_WAIT_DOWN = 1;
elseif strcmp(callback_arg, 'up')
  if VERBOSE, fprintf('u'); end
  VGG_EVENT_WAIT_E = [p button -1];
  VGG_EVENT_WAIT_DOWN = 0;
elseif strcmp(callback_arg, 'key')
  if VERBOSE, fprintf('k'); end
  key = get(gcf,'CurrentCharacter');
  if any(abs(key))
    VGG_EVENT_WAIT_E = [p abs(key) 2];
  end
end
set(VGG_EVENT_WAIT_HANDLE, 'userdata', 1);
```

Table S1. Absolute values of the SMDs for oral processing. Values are provided for each food pair in the low and high energy-dense subgroups, separately. Respectively, *L and *H indicates that the test meal belongs to the low- and high- energy dense subgroup.

Subgroup	Food Pair	Eating Rate (kcal/s)	Initial Eating Rate (g/s)	Change in Eating Rate (g/s^2)	Oral Sensory Exposure Time (s)	Interbite Interval (s)	Average Bite Size (g)	Bite Rate (Per Second)	Number of Chews (Per Mouthful)	Total
*L	Fish pie *vs.* Vegetable biryani	0.9	0.1	1.1	1.0	0.6	1.2	0.0	0.9	5.7
*L	Mushroom risotto *vs.* Vegetable biryani	1.0	0.0	0.8	1.0	0.6	1.0	0.2	0.7	5.3
*L	Chicken in barbeque sauce with wedges *vs.* Vegetable biryani	0.8	0.2	0.7	1.0	0.4	1.0	0.3	0.6	5.0
*L	Roast chicken dinner *vs.* Vegetable biryani	0.3	0.6	1.4	0.6	0.6	0.3	0.1	0.8	4.7
*L	Roast chicken dinner *vs.* Chicken in barbeque sauce with wedges	0.6	0.4	0.4	0.5	0.4	0.5	0.4	0.2	3.5
*L	Roast chicken dinner *vs.* Fish pie	0.7	0.7	0.2	0.5	0.1	0.7	0.1	0.2	3.1
*L	Roast chicken dinner *vs.* Mushroom risotto	0.9	0.5	0.2	0.6	0.1	0.5	0.1	0.1	2.9
*L	Chicken in barbeque sauce with wedges *vs.* Mushroom risotto	0.5	0.2	0.4	0.2	0.3	0.0	0.5	0.2	2.3
*L	Chicken in barbeque sauce with wedges *vs.* Fish pie	0.2	0.4	0.5	0.1	0.3	0.2	0.2	0.4	2.3
*L	Fish pie *vs.* mushroom risotto	0.3	0.0	0.1	0.1	0.0	0.3	0.2	0.1	1.1
*H	Beef stew with dumplings *vs.* Fish, chips and peas	2.3	1.3	0.5	1.8	0.8	2.5	0.1	0.5	9.9
*H	Beef lasagne *vs.* Fish, chips and peas	2.0	0.9	0.4	1.3	0.2	2.1	0.1	0.6	7.5
*H	Chicken and bacon pasta bake *vs.* Fish, chips and peas	1.9	1.3	0.7	1.2	0.6	1.3	0.3	0.2	7.4
*H	Beef cannelloni *vs.* Fish, chips and peas	1.8	1.2	0.4	1.4	0.0	1.5	0.0	0.7	7.0
*H	Beef cannelloni *vs.* Beef stew with dumplings	0.8	0.6	0.6	0.4	0.9	0.6	0.1	0.2	4.2
*H	Beef lasagne *vs.* Beef stew with Dumplings	0.5	0.6	0.7	0.2	1.0	0.8	0.1	0.2	4.1
*H	Beef stew with dumplings *vs.* Chicken and bacon pasta bake	0.8	0.6	0.7	0.5	0.3	0.8	0.1	0.2	4.1
*H	Beef cannelloni *vs.* Chicken and bacon pasta bake	0.1	0.0	0.2	0.1	0.7	0.2	0.2	0.3	1.9
*H	Beef lasagne *vs.* Chicken and bacon and pasta bake	0.2	0.1	0.0	0.2	0.7	0.2	0.2	0.3	1.9
*H	Beef cannelloni *vs.* Beef lasagne	0.3	0.0	0.2	0.1	0.2	0.1	0.0	0.0	0.8
